# Conserved upstream open reading frames in higher plants

**DOI:** 10.1186/1471-2164-9-361

**Published:** 2008-07-31

**Authors:** Michael K Tran, Carolyn J Schultz, Ute Baumann

**Affiliations:** 1 Australian Centre for Plant Functional Genomics PMB 1 Glen Osmond SA 5064, Australia; 2 School of Agriculture, Food, and Wine, University of Adelaide, Waite Campus, Glen Osmond, SA 5064, Australia

## Abstract

**Background:**

Upstream open reading frames (uORFs) can down-regulate the translation of the main open reading frame (mORF) through two broad mechanisms: ribosomal stalling and reducing reinitiation efficiency. In distantly related plants, such as rice and Arabidopsis, it has been found that conserved uORFs are rare in these transcriptomes with approximately 100 loci. It is unclear how prevalent conserved uORFs are in closely related plants.

**Results:**

We used a homology-based approach to identify conserved uORFs in five cereals (monocots) that could potentially regulate translation. Our approach used a modified reciprocal best hit method to identify putative orthologous sequences that were then analysed by a comparative R-nomics program called uORFSCAN to find conserved uORFs.

**Conclusion:**

This research identified new genes that may be controlled at the level of translation by conserved uORFs. We report that conserved uORFs are rare (<150 loci contain them) in cereal transcriptomes, are generally short (less than 100 nt), highly conserved (50% median amino acid sequence similarity), position independent in their 5'-UTRs, and their start codon context and the usage of rare codons for translation does not appear to be important.

## Background

RNA-omics, or more simply R-nomics, is the large-scale study of RNA structure and function [1]. One of the major challenges faced by R-nomics is to understand the regulatory mechanisms of complex signals found in the untranslated regions (UTRs) of messenger RNAs. In particular, the control signals found in the 5'-UTR of some eukaryotic mRNAs play a crucial role in translational control that can result in rapid changes to the proteome [2]. These post-transcriptionally regulated mRNAs frequently encode important regulatory proteins (e.g., proto-oncogenes, growth factors, and transcription factors) [3] that need to be strongly or precisely regulated for normal cellular activity. In other cases, control signals in the 5'-UTR provide continuous regulation of essential mRNAs by providing an alternative route for translation when cap-dependent translation is compromised (e.g., under stress conditions) [4].

Translational control signals are often found in long 5'-UTRs (>100 nt) [5] where they can contain either a single control signal [6] or multiple control signals that function independently [7] or in a coordinated fashion [8-10]. One important translational control signal found in both prokaryotes and eukaryotes is the upstream open reading frame (uORF), a small open reading frame located upstream of the main coding region [11].

Two types of functional upstream open reading frames have been described that have a demonstrated activity either *in-vitro *or *in-vivo*: a) uORFs encoding bioactive peptides [12-15] that either act on translation or have biological roles other than reducing the translation of the main ORF, and therefore can be described as sequence-dependent, and b) sequence-independent uORFs. A sequence-dependent uORF encodes a small peptide, and some of these uORF-encoded peptides have been shown to directly affect translation via either ribosomal stalling during translation of the uORF or termination of translation by inhibiting the peptidyl transferase activity of the ribosome and thus peptide bond formation [16,17]. For sequence-independent uORFs, the uORF-encoded peptide is not important for translational control, but other factors like uORF recognition, length, stop codon environment, and the downstream intercistronic sequence (length and structure) can affect reinitiation efficiency at the downstream ORF [18,19]. Sequence-independent uORFs can also indirectly affect translation by allowing ribosomes to bypass inhibitory stem structures [20] or activate dormant internal ribosome entry sites (IRES) [8] via conformational changes induced by the translation of the uORF. These distinct mechanisms of translational control have been proven to be important through *in-vitro *genetic (mutational analyses) and biochemical (toe-printing) assays [16].

There are two known pathways where uORFs can influence mRNA stability. Studies in yeast have indicated that both sequence-dependent and sequence-independent uORFs can cause mRNA destabilisation by the nonsense-mediated mRNA decay pathway [21]. Mutations in the mRNA 5'-UTR that insert an uORF trigger the nonsense-mediated decay pathway and lead to decapping of the mRNA. Alternatively, mRNA destabilisation can occur via the termination dependent decay pathway [22]. In this pathway, the 40S ribosomal units are released from the mRNA due to features such as stop codon environment (e.g., GC rich) or short intercistronic sequence containing a secondary structure. Release of the 40S ribosomal units prevent reinitiation of translation downstream of the uORF, and the mRNA becomes susceptible to decay. The mechanisms underlying both uORF nonsense-mediated decay and post-termination mediated decay remain unclear.

Identifying uORFs involved in regulation of gene expression remains a challenge [16,23,24]. Recently it has been estimated that it would take 20 man-months to find a single functional uORF by random selection and testing of yeast mRNAs [25]. To overcome this problem Selpi *et al*. [25] used an artificial intelligence approach called inductive logic programming to identify likely functional uORFs. The approach used rules based on background knowledge of uORFs in yeast mRNAs and as such may not be applicable to other organisms such as plants.

Another approach for identifying sequence-independent uORFs was recently described [26]. Kochetov *et al*. [26] selected human mRNAs with specific sequence organisation (i.e., uORF overlapping the main ORF) that could facilitate reinitiation at downstream start codons. If the downstream start codons were nested in-frame with the main ORF then potentially N-terminally truncated variants of the main protein could be produced via reinitiation. Kochetov *et al*. [26] reported that 297 out of 754 mRNAs (39% of the sub-sample) contained this specific sequence organisation with an average intercistronic spacer of 66 ± 77 nt, which provides sufficient space for reinitiation. This novel approach highlights another way in which uORFs can be functional via the generation of novel protein isoforms.

The number of characterised uORFs in plants is apparently less than 100 (0.3%) based on a PubMed search, and about four have been identified in cereals. They include the uORFs of the S-adenosylmethionine decarboxylase gene (AdoMetDC) in both monocots and dicots [9,27,28], rice myb7 gene [29]; transcription factorssuch as maize Opaque-2 [30], maize *R *[31], and maize *Lc *[7]. Also, uORFs have been found in dicot plant genes that include *AtB2/AtbZIP11 *[32], *ABI3 *[33], and *CpbZIP2 *[34]; and auxin responsive factor genes *ETT *and *MP *[35]. These characterised uORFs (<0.3%) in plants are much lower than the estimated number of genes that contain uORFs, which can vary from 11% [36] to 60% [12].

One strategy for identifying functional uORFs in plants is to use a comparative approach [12,13,37]. There are extensive assembled EST datasets for five important cereal crops and Arabidopsis. The cereals include rice (*Oryza sativa *L.), wheat (*Triticum aestivum*), barley (*Hordeum vulgare*), maize (*Zea mays*), and sorghum (*Sorghum bicolor*). Rice is the best characterised of these cereals with a sequenced genome [38] and a cDNA database containing 32,000 clones that were enriched for 5' full-length sequences [39]. Cereals such as wheat are unlikely to be fully sequenced in the near future because of their large genome size. Wheat has a hexaploid genome of 16000 Mb [40] that is 37 times that of rice (430 Mb), and 5.5 times (2900 Mb) the size of the human genome [41]. A comparative approach is likely to identify sequence dependent uORFs [16] where the encoded peptide of the uORF is involved in regulation of gene expression.

In this study, we used comparative R-nomics to identify conserved uORF motifs in cereals and Arabidopsis. We constructed a bioinformatics pipeline called uORFSCAN that performs a comparative analysis on the important agronomic crops rice, wheat, barley, maize, and sorghum; and the well studied dicot plant Arabidopsis. To account for the variable quality of assembled EST data, we have used orthologous sequence clustering, iterative sequence analysis, and manual curation. Our comparative method is easily transferable to uORF identification in other species.

## Methods

### Data material

KOME (Knowledge-based Oryza Molecular biological Encyclopedia) full-length rice cDNA sequences were obtained from . This file is dated Tuesday, 24 January 2006, and contains 32,127 full-length cDNA clones (originally 28,469). The TIGR plant gene indices database  was used to obtain tentative contigs (TCs) from wheat (release 10.0, Jan 05, 580155 ESTs, 44954 TCs), barley (release 9.0, Sept 04, 370546 ESTs, 23176 TCs), maize (release 17.0, Nov 06, 695811 ESTs, 56687 TCs), and sorghum (release 8.0, Nov 05, 187282 ESTs, 20029 TCs). Data cleaning was performed on the TIGR dataset to select for sequences that are designated as tentative contigs (identifiers prefixed with "TC"), thereby excluding all singletons. All data files were imported and managed using Microsoft Access 2003. We also re-ran the analysis using the TIGR Plant Transcript Assemblies (last updated on October 17^th^, 2006) for wheat (840871 ESTs), barley (456410 ESTs), maize (1084701 ESTs), and sorghum (203575 ESTs) on the uORFSCAN pipeline, but did not find any additional conserved upstream open reading frames (uORFs).

### Orthologue searches

The reciprocal best hit method (rbh) was adapted to account for alternative splice forms that are present in the KOME dataset that would otherwise give many false negatives. The problem with alternative splice forms is that they will never have the highest score in the reverse BLAST because the presence of a longer alternative splice form will always be listed higher on the hit list due to the way BLAST [42] ranks hits (according to score and e-value). To account for alternative splice forms, we examined not only the top hit but also similar hits (percent identity to top hit: Δ -5%, similar length to top hit: +/- 20%) for symmetry with the top hit in the forward blast. If there is symmetry between the forward and reverse blasts then we considered the reciprocal pair to be orthologous. General parameters for similarity searches were: tblastx program, expect threshold value at 1.0e-50, scoring parameters, BLOSUM62 matrix; gap costs (existence, 11; extension 1), and filter and masking, off. Only sequence alignments with at least 70% sequence coverage were considered further. Similarity searches were performed at The South Australian Partnership for Advanced Computing (SAPAC) .

### Verification of main ORF

The rice cDNA sequences containing conserved uORFs were used in a blastn search against NCBI Non-redundant database to identify uORFs predicted from ribosomal RNA genes, chloroplastic genes, and mitochondrial genes. These genes do not represent coding genes derived from the nuclear genome, and therefore have been removed from this study. Also, the main open reading frames, predicted by uORFSCAN were used to search (blastn) the coding sequence (CDS) annotations from TIGR rice pseudomolecules database . Alignments not starting from the beginning of the CDS were regarded as suspicious. As additional verification, the rice main open reading frame predictions were also compared with protein data from The UniProt Knowledgebase (UniProtKB) . Translations of the rice cDNA sequences in the same frame as the predicted main open reading frame, starting from the 5'-untranslated region to the end of the main open reading frame, were used to search (blastp) against UniProtKB. Aligments not beginning from the start of the protein sequence were discarded if they also did not have TIGR CDS support.

### Statistical analysis of codon usage

The *p-values *were calculated according to the following formulas:

The probability to observe the number of times each codon was present in the uORFs (n_obs_) that was less than or equal to the expected (n_av_) by chance alone is:

(1) $$P=\sum_{\text{n}=0}^{{\text{n}}_{\text{obs}}}\left(\begin{array}{c} \text{N}\\ \text{n} \end{array}\right)P^{\text{n}}{\left(1-P\right)}^{\text{N-n}}$$

The probability to observe the number of times each codon was present in the uORFs (n_obs_) that was greater than or equal to the expected (n_av_) by chance alone is:

(2) $$P=\sum_{\text{n}={\text{n}}_{\text{obs}}}^{\text{N}}\left(\begin{array}{c} \text{N}\\ \text{n} \end{array}\right)P^{\text{n}}{\left(1-P\right)}^{\text{N-n}}$$

Where,

n_obs _= The observed number of times a codon was present in the uORFs.

n_av _= The average number of times a codon was present in the uORFs based on the frequency of this codon in the mORF and the sample size (the observed number of codons for the set of codons for an amino in the uORFs).

## Results

### The uORFSCAN pipeline for discovering uORFs

**Figure 1:**
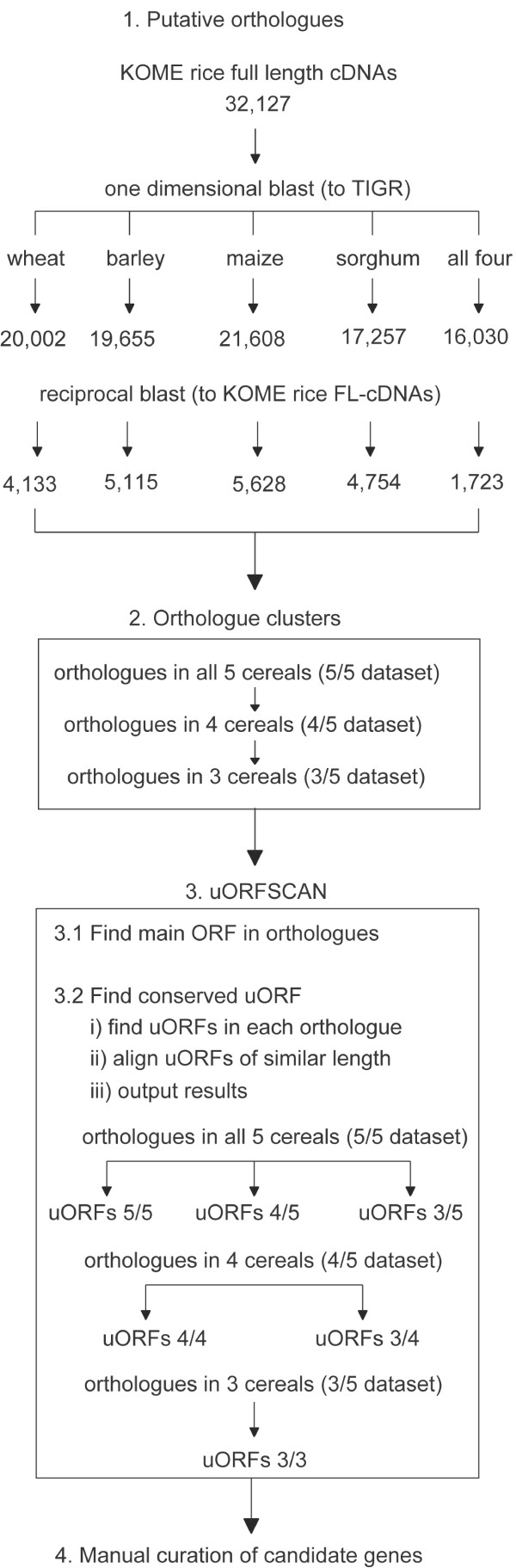
**Tran_Figure1.eps**. 'Overview of the uORFSCAN pipeline'. The pipeline consists of four steps: 1) Identifying putative orthologues using a modified reciprocal best hit (rbh) method, 2) Clustering of orthologues according to how many cereal species they are found in, 3) Using uORFSCAN program to find conserved uORFs using a comparative approach, and 4) manual curation of predicted conserved cereal and Arabidopsis uORFs.

The uORFSCAN pipeline used rice full-length cDNAs [39] and wheat, barley, maize, and sorghum assembled EST data for comparative analysis (Figure 1). In the first step of the pipeline, we identified rice genes that had orthologues in wheat, barley, maize, and sorghum. The use of orthologous sequences allowed us to more accurately predict the main coding region and define the 5'-UTR that is necessary to identify conserved uORFs.

The reciprocal best hit (rbh) method was used to find true orthologues by a process of eliminating paralogues [43,44]. The principle of rbh is that a pair of sequences are orthologues if they are each others best hit. We modified the rbh method to find orthologues of rice genes in wheat, barley, maize, and sorghum such that it allowed us to keep alternative splice forms while at the same time eliminating paralogues. Alternative splice forms of a gene were distinguished by changes in gene length while still maintaining high sequence identity. We found that the modified reciprocal best hit method eliminated 70–75% of paralogue sequences. For example, in the one directional BLAST against the barley assembled EST database 19,655 sequences were identified, however this number was reduced to 5,115 (26%) sequences when the reciprocal best hit method was used (Figure 1, Step 1).

Only 1723 of the rice genes had conserved orthologues in the other four cereals (wheat, barley, maize, and sorghum), most likely because none of the assembled EST datasets contained the entire transcriptome. To account for missing or erroneous sequences, we grouped the orthologues into three datasets for 5'-UTR analysis (Figure 1, Step 2). The datasets included rice genes that had orthologues in four other cereals (5 out of 5 dataset), in three other cereals (4 out of 5 dataset), and in two other cereals (3 out of 5 dataset).

In Figure 1 (Step 3), we developed a program called uORFSCAN (see Additional file 9)  to find conserved uORFs. uORFSCAN takes as input a FASTA file containing the rice cDNA sequence and its orthologues, and identifies for each of these sequences all the possible open reading frames (ORFs). In the first iteration, the longest conserved ORF was designated as the main coding region. However, the longest ORF is not always the main coding region when there are other ORFs of similar length. Therefore, a comparative approach was used to identify the main coding region (Figure 1, Step 3.1). This involved finding the longest ORF that was present in all orthologous sequences, and then iteratively reducing the number of orthologous sequences, one at a time, to determine if a longer conserved set of ORFs could be found, and finally terminating when there was no improvement. The longest ORF in at least three out of five cereals was considered the main coding region. In Figure 1 (Step 3.2), uORFSCAN attempts to align rice uORFs with similar length orthologous uORFs (+/- 5%) at the protein level using ClustalW ([45], see Additional file 8) . Finally, uORFSCAN analysed each alignment file to determine the average conservation of the uORFs, and grouped the alignments based on the number of conserved orthologous uORFs found. For example, using the 4 out of 5 dataset generated the 4 out of 4 and the 3 out of 4 datasets (Figure 1). We report uORFs from orthologous genes that shared sequence similarity because of our interest in finding functional uORFs.

The final step (Figure 1, Step 4) was manual curation to verify the predicted rice main coding region of each gene by comparing it with the genome annotation and other protein data. This was necessary, as uORFSCAN is expected to be sensitive to inaccurate (e.g., frame-shifts) and/or incomplete sequence data. For example, rice full length sequences can be incomplete because of failure of the 5' capping method [39]. If the coding region is truncated, this can result in an internal methionine selected as the start codon and therefore the derived 5'-UTR is actually coding sequence, which is often highly conserved and can lead to false positive predictions.

### Conserved upstream open reading frames appear to be rare

The uORFSCAN pipeline identified nine cDNAs containing uORFs that were conserved in the five out of five (5/5) dataset containing orthologous sequences in all 5 cereals (Table 1). Three of these cDNAs encoded multiple uORFs, one of the cDNAs being AdoMetDC, which has previously been reported to contain two uORFs [46]. We manually curated all nine cDNAs and showed that they were all reliable based on our validation criteria (Table 2), which included the removal of the uORFs predicted from ribosomal rRNA genes (data not shown). The cDNAs included the multiple uORFs in S-adenosylmethionine decarboxylase cDNA [46], alkaline phytoceramidase cDNA, calcineurin B-like (CBL)-interacting protein kinase cDNA; and a single conserved uORF in a cDNA encoding an oxidoreductase protein, ribosomal protein S6 kinase, trehalose-6-phosphate phosphatase, ubiquitin-fold protein, F9L1.29 protein, and an ankyrin-3 protein.

**Table 1:** The uORFs predicted by uORFSCAN in 5 out of 5

Rice		Wheat		Barley		Maize		Sorghum		Avg. A.A. similarity (%)	Putative function^b^
		
Identifier	5'-UTR	Identifier	5'-UTR^a^	Identifier	5'-UTR^a^	Identifier	5'-UTR^a^	Identifier	5'-UTR^a^		
AK106095	131_9_17	TC265929	113_9_16	TC148181	67_9_16	TC288369	131_9_17	TC102998	149_9_17	100	Oxidoreductase
AK103391^c^	205_75_74	TC269775	251_75_62	TC134190	204_75_62	TC294011	215_75_75	TC103599	106_75_378	88	Trehalose-6-phosphate phosphatase
AK100589 ^d,e^	240_9_334	TC264559	201_9_317	TC130707	228_9_318	TC292591	286_9_320	TC91317	260_9_329	50	S-adenosylmethionine decarboxylase (AdoMetDC)
	248_156_179		209_150_168		236_150_169		294_153_168		268_153_177	90	
	296_108_179		254_105_168		281_105_169		336_111_168		310_111_177	92	
AK073303	67_9_142	TC237149	75_9_113	TC132556	81_9_139	TC305609	127_9_69	TC102988	222_9_69	50	Alkaline phytoceramidase
	135_9_74		75_9_113		81_9_139		127_9_69		222_9_69	50	
AK072868 ^e^	249_27_248	TC247418	258_27_266	TC139536	298_27_272	TC306591	444_27_564	TC102544	331_27_265	11	CBL-interacting protein kinase
	259_195_70		268_198_85		308_198_91		260_192_583		341_195_87	29	
	269_39_216		278_39_234		318_39_240		768_39_228		351_39_233	8	
	338_90_96		347_93_111		387_93_117		576_93_366		420_90_113	10	
	392_36_96		404_36_111		444_36_117		633_36_366		474_36_113	8	
AK072649	100_192_117	TC236348	79_192_117	TC133316	76_192_93	TC305793	180_192_116	TC93140	168_192_116	78	Ribosomal protein S6 kinase
AK066145	178_12_58	TC266262	149_12_73	TC134484	154_12_231	TC286452	224_12_70	TC94546	187_12_69	33	Ubiquitin-fold protein
AK064792	276_15_187	TC267323	254_15_188	TC132983	253_15_-9	TC306152	263_15_170	TC107743	230_15_150	87	F9L1.29 protein
AK060523	173_123_185	TC235416	201_126_157	TC148319	211_120_163	TC305149	255_129_195	TC103609	240_129_212	58	Ankyrin-3

^a ^Pre-orf distance_uORF length_intercistronic distance.

^b ^Functional annotation based on "The UniProt Knowledgebase (UniProt)" database.

^c ^One of several genes (identifiers) that are in multiple tables because different conserved uORFs were identified in the different datasets.

^d ^Previously reported upstream open reading frames (see 'Introduction' section on AdoMetDC).

^e ^Contain one or more nested uORFs.

Ribosomal rRNA genes have been removed.

See Table 2 for criteria for verifying rice uORFs in 5 out of 5.

**Table 2:** Criteria for verifying rice uORFs in 5 out of 5

Accession	FL- cDNA^a^	Upstream & In-frame stop codon	Agreement with genome annotation^b^	Alignment of uORFSCAN identified main proteins with UniProt proteins^c^						uORF valid
					
				UniProt protein length (AA)	Align length (AA)	Identities (%)	Expect	Annotation	GO classication *(Arabidopsis thaliana)*	
AK106095	Yes	Yes	Yes	392	392	100	2.2e-217	Oxidoreductase	[go:19538] protein metabolism [go:16706] oxidoreductase activity	Yes
AK103391	Yes	Yes	Yes	371	371	100	3.4e-194	Trehalose-6-phosphate phosphatase	[go:5992] trehalose biosynthesis [go:9507] chloroplast [go:4805] trehalose-phosphatase activity	Yes
AK100589	Yes	Yes	Yes	398	398	100	1.1e-215	AdoMetDC	[go:6596] polyamine biosynthesis [go:5694] chromosome	Yes
AK073303	Yes	Yes	Yes	257	257	100	1.6e-141	Acyl-CoA independent ceramide synthase	[go:6672] ceramide metabolism [go:16020] membrane [go:3824] catalytic activity [go:16811] hydrolase activity	Yes
AK072868	Yes	Yes	Yes	443	443	100	3.6e-238	uncharacterized protein (probable CBL-interacting serine/threonine-protein kinase 15)	[go:6468] protein phosphorylation [go:7165] signal transduction [go:5524] ATP binding [go:4672] protein kinase activity	Yes
AK072649	Yes	Yes	Yes	480	488	76	9.6e-199	Ribosomal protein S6 Kinase	[go:45946] positive translation [go:6468] protein phosphorylation [go:9507] chloroplast [go:16301] kinase activity	Yes
AK066145	No	Yes	Yes	119	119	100	1.3e-59	Membrane-anchored ubiquitin-fold protein	[go:6464] protein modification	Yes
AK064792	Yes	Yes	197^d^	109	108	57	8.4e-26	F9L1.29 protein	Not available	Yes
AK060523	Yes	Yes	Yes	166	166	100	1.9e-88	uncharacterized protein (probable ankyrin-3)	[go:9507] chloroplast [go:5515] protein binding	Yes

^a ^Used rice cDNA in blastn search against "NCBI EST_Others" database (rice) to search for longer 5' ESTs.

^b ^Used rice cDNA in blastn search against "TIGR Rice Genome Annotation DB: Coding Sequences" database to verify the cDNA ORF.

^c ^Translated the rice cDNA in the same frame as the main open reading frame identified by uORFSCAN (include translations upstream of predicted start Methionine). The resulting protein sequence was used in a blastp search against "The UniProt Knowledgebase (UniProt)" database.

^d ^The genome annotation for the CDS is longer by the indicated number of base pairs.

To account for variable quality in assembled EST data, we also looked for cases where the uORFs (4/5, 3/5, 4/4, 3/4, and 3/3 result set) were conserved in only four out of five (4/5) orthologues and three out of five orthologues (see Additional files 1, 2, 3, 4, 5; Figure 1, Step 3.2). In brief, the 4/5 result set contains 16 rice genes with a total of 20 conserved uORFs in orthologous cereal genes, the 3/5 result set contains 44 rice genes with a total of 79 conserved uORFs in orthologous cereal genes, the 4/4 result set contains 16 rice genes with a total of 23 conserved uORFs in orthologous genes, the 3/4 result set contains 113 rice genes with a total of 129 conserved uORFs in orthologous genes, and finally the 3/5 result set contains 65 genes with a total of 93 conserved uORFs in orthologous genes.

In order to identify sequence dependent uORFs, we extended our search for cereal uORFs that might also be conserved in the dicot Arabidopsis by using the rice cDNAs that contained conserved uORFs in four other cereals (5/5 result set) and the Arabidopsis Tair 7 cDNA dataset (see Methods). The uORFSCAN pipeline identified 13 rice cDNAs containing uORFs that were conserved in Arabidopsis (Table 3). Four of these cDNAs encoded multiple uORFs. Of the 13 cDNAs with uORFs, only 11 were verified as reliable based on manual curation (Table 4) that removed the uORFs predicted from a cDNA encoding a helicase. Manual curation of the helicase cDNA revealed that the genome and protein annotation for the coding region extended further upstream than predicted by uORFSCAN, highlighting the limitations of using assembled EST data where frame-shift errors was the likely reason for the false positive prediction. The reliable predictions included the multiple uORFs found in a cDNA encoding ww domain containing protein, trehalose-6-phosphate phosphatase, GAMYB-binding protein, and ankyrin-3. The latter three cDNAs contained a combination of uORFs that were conserved between the cereals (rice and at least two other cereals) and Arabidopsis, and uORFs conserved between rice and Arabidopsis (Table 3). uORFSCAN also identified seven rice cDNAs containing a single uORF that were conserved in Arabidopsis and in almost all cases (except cDNA encoding an auxilin-like protein) the cereals as well (Table 3). They included the uORFs found in a cDNA encoding phosphatase 2a protein, homeodomain containing protein, S-Adenosylmethionine decarboxylase, auxilin-like protein, CBL-interacting protein kinase, protein kinase ATN1, and a hypothetical protein.

**Table 3:** Rice uORFs predicted by uORFSCAN that are conserved in Arabidopsis

Rice		Arabidopsis		Avg. A.A. similarity (%)	Putative function^b^
			
Identifier	5'-UTR^a^	Identifier	5'-UTR^a^		
AK101100	142_12_21^c,d^	AT1G51690.1	555_12_1160	33	Protein phosphatase 2a
AK066952	365_66_182	AT3G13225.1	364_63_431	27	WW domain containing protein
	368_63_182^e^		364_63_431	29	
	503_51_59		553_51_254	1	
AK119592	304_90_148^c,d^	AT3G01470.1	162_87_120	36	Homeodomain leucine zipper protein
AK100589	248_156_179^c,d^	AT3G02470.3	222_156_154	82	S-Adenosylmethionine decarboxylase
AK103391	176_30_148^c,d,f^	AT4G22590.1	254_30_137	44	Trehalose-6-phosphate phosphatase
	205_75_74^g^		283_75_63^g^	71	
AK069534	813_9_432	AT4G12770.1	41_9_108	50	Auxilin-like protein
AK069526	214_126_544^c^	AT4G19110.2	255_126_527	44	GAMYB-binding protein
	690_9_185^c^		603_9_296	50	
	820_36_28		398_36_474	17	
AK072868	338_90_96^c,d^	AT5G58380.1	11_87_295	17	CBL-interacting protein kinase
AK060523	173_123_185^c^	AT5G07840.1	289_117_250	36	Ankyrin-3
			313_93_250^e^	44	
	206_90_185^e^		313_93_250^e^	33	
AK067412	222_84_49^c,h^	AT5G50180.1	357_84_79	4	Protein kinase ATN1
AK102277	228_117_150^c^	AT1G68550.1	309_96_95	21	Hypothetical protein
*AK100332*	*1174_21_883*	*AT5G44800.1*	*359_21_3*	*14*	*Helicase*
	*1618_21_439*		*359_21_3*	*17*	
	*1810_21_247*		*359_21_3*	*17*	
*AK059639*	*1_45_784*^c^	*ATCG00920.1*	*55_45_844*	*86*	*40S ribosomal protein S15*

^a ^Pre-uORF distance_uORF length_intercistronic distance.

^b ^Functional annotation based on "the UniProt Knowledgebase (uniProt)" database.

^c ^Rice uORF is conserved in at least two orthologous cereal and Arabidopsis genes.

^d ^Rich in serine (at least 20%).

^e ^Nested uORF.

^f ^One of several genes (identifiers) that are in multiple tables because different conserved uORFs were identified in the different datasets.

^g ^Overlapping uORF.

^h ^Rice in arginine (at least 20%).

Ribosomal rRNA genes have been removed.

Rows in italics are false positive predictions (see Table 4. Criteria for verifying rice uORFs that are conserved in Arabidopsis)

**Table 4:** Criteria for verifying rice uORFs that are conserved in Arabidopsis

Accession	FL- cDNA^a^	Upstream & In-frame stop codon	Agreement with genome annotation^b^	Alignment of uORFSCAN identified main proteins with UniProt proteins^c^						uORF valid
					
				UniProt protein length (AA)	Align length (AA)	Identities (%)	Expect	Annotation	GO classication *(Arabidopsis thaliana)*	
AK101100	Yes	Yes	Yes	525	525	100	5.0e-287	Protein phosphatase 2A	[go:6470] protein dephosphorylation [go:166] nucleotide binding	Yes
AK066952	Yes	Yes	Yes	860	694	99	0	WW domain containing protein	Not available	Yes^d^
AK119592	Yes	Yes	Yes	343	343	100	6.8e-187	Homeodomain leucine zipper protein	[go:6355] regulation of transcription [go:3677] DNA binding	Yes
AK100589	Yes	Yes	Yes	398	398	100	1.1e-215	S-Adenosylmethionine decarboxylase	[go:6596] polyamine biosynthesis [go:5694] chromosome	Yes
AK103391	Yes	Yes	Yes	371	371	100	3.3e-194	Trehalose-6-phosphate phosphatase	[go:5992] trehalose biosynthesis [go:9507] chloroplast	Yes
AK069534	Yes	Yes	1066^e^	485	413	61	7.6e-117	Auxilin-like protein	Not available	Yes^f^
AK069526	Yes	Yes	Yes	483	483	83	5.8e-256	GAMYB-binding protein	[go:6468] protein phosphorylation [go:5524] ATP binding [go:16301] kinase activity	Yes
AK072868	Yes	Yes	Yes	443	443	100	3.5e-238	CBL-interacting kinase 15	[go:6468] protein phosphorylation [go:5524] ATP binding [go:16301] kinase activity	Yes
AK060523	No	Yes	Yes	166	166	99	8.2e-88	Ankyrin-3	[go:5515] protein binding	Yes
AK067412	Yes	Yes	Yes	353	353	72	1.2e-136	Protein kinase ATN1	[go:6468] protein phosphorylation [go:5524] ATP binding [go:16301] kinase activity	Yes
AK102277	Yes	Yes	Yes	338	338	99	4.9e-179	Hypothetical protein	Not available	Yes
AK100332	Yes	Yes	4092^e^	2192	872	30	5.3e-28	Helicase	[go:3676] nucleic acid binding [go:6355] regulation of transcription [go:5515] protein binding	No^g^
AK059639	No	Yes	Yes	154	154	100	2.6e-77	40S ribosomal s15 protein	[go:3735] structural part of ribosome [go:6412] protein biosynthesis	No^h^

^a ^Used rice cDNA in blastn search against "NCBI EST_Others" database (rice) to search for longer 5' ESTs.

^b ^Used rice cDNA in blastn search against "TIGR Rice Genome Annotation DB: Coding Sequences" database to verify the cDNA ORF.

^c ^Translated the rice cDNA in the same frame as the main open reading frame identified by uORFSCAN (include translations upstream of predicted start Methionine). The resulting protein sequence was used in a blastp search against "The UniProt Knowledgebase (UniProt)" database.

^d ^The protein data suggests that the main open reading frame predicted by uORFSCAN extends further upstream, but does not overlap the predicted uORFs and so the uORFs are still valid.

^e ^The genome annotation for the CDS is longer by the indicated number of base pairs.

^f ^A shorter protein was identified, but does not overlap the predicted uORFs and so the uORFs are still valid.

^g ^A longer protein was identified indicating the main open reading frame extends further upstream, and does overlap the predicted uORFs and so the uORFs are not valid.

^h ^Possibly not functional because pre-orf distance is less than 20 nucleotides that is thought to be required for translation initiation.

### Position and occupation of uORFs in 5'-UTRs

**Figure 2:**
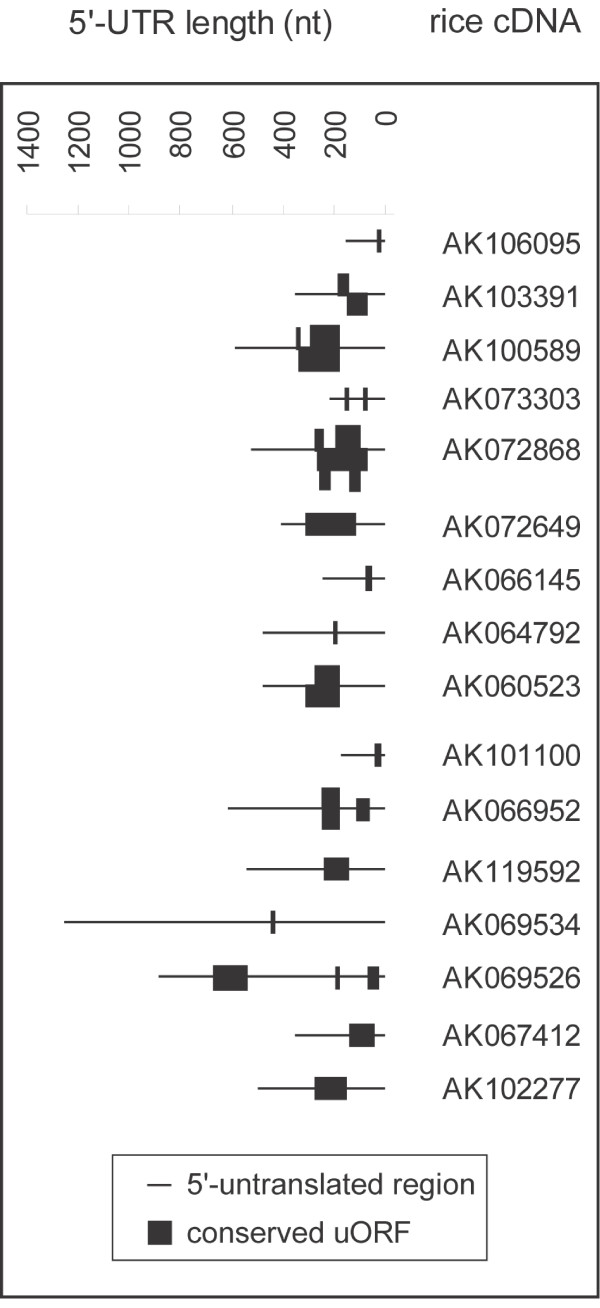
**Tran_Figure2.eps**. 'The position of conserved uORFs within their 5'-UTRs'. It contains rice uORFs conserved in four other cereals and in Arabidopsis.

Studies have shown that the position of an uORF within its 5'-UTR, which determines the pre-orf and intercistronic distances, can have profound effects on its function [18,22]. When we examined the positions of cereal uORFs within their 5'-UTRs we found that there was no positional preference with the exception that they were not positioned too closely to the start of their individual 5'-UTR and coding region (Figure 2). For example, all of the uORFs conserved in five orthologous cereals (5/5 result set) and in Arabidopsis were at least positioned 20 nucleotides from the start of their 5'-UTR, which is thought to be the minimum number of nucleotides required for a functional uORF [18]. We noticed that the intercistronic distances for these uORFs were generally shorter than the pre-orf distance. We also found seven uORFs, which included the functional small AdoMetDC uORF, that occupied greater than 20% of their individual 5'-UTR.

### The length distribution of uORFs

**Figure 3:**
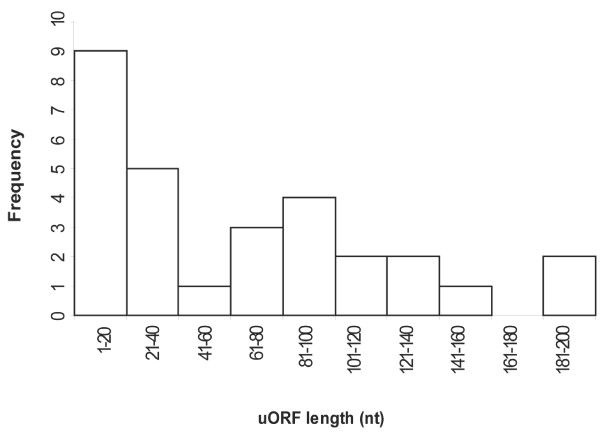
**Tran_Figure3.eps**. 'A frequency distribution of the length (nt) of rice uORFs conserved in four other cereals and in Arabidopsis.

Since earlier reports showed that plant uORFs can vary in length from 6 to 156 nucleotides [7,9,29-31,46], we examined the length distribution of the cereal uORFs. There are two peaks in the distribution that were found between 1 to 40 nucleotides, and 81 to 120 nucleotides (Figure 3). The uORFs found in the first peak are tiny with 9 (out of 14) uORFs having a length of nine nucleotides. Some of these tiny uORFs could be artefactual as a result of point mutations that insert an in-frame start and/or stop codon in the 5'-UTR. If these artefactual uORFs were removed then the uORF length distribution would move towards a normal distribution. Seventy six percent of the uORFs in the length distribution are shorter than 100 nucleotides, and 48% are shorter than 40 nucleotides. The shortest conserved uORF found in four independent cDNAs was nine nucleotides, even though the cut-off length used by uORFSCAN to identify uORFs was six nucleotides (a start and a stop codon). One of the nine nucleotide uORFs was the 5' tiny uORF found in the S-adenosylmethionine decarboxylase cDNA [9], and three new uORFs, two found in a cDNA encoding alkaline phytoceramidase, and one in a cDNA encoding oxidoreductase, (Table 1). Two long conserved uORFs (>181 nucleotides) were found in cDNAs encoding protein kinases that included one uORF found in a cDNA encoding a CBL-interacting protein kinase and another uORF found in a cDNA encoding a ribosomal protein S6 kinase.

### Sequence conservation in uORFs

The level of amino acid sequence conservation in cereal uORFs was generally high, as expected, based on our approach of reporting similar length orthologous uORFs that shared sequence similarity. For example, in the 5 out of 5 dataset the median value is 50% sequence similarity. When the two main datasets were included (uORFs conserved in all five cereals and uORFs conserved between rice and Arabidopsis), the median value is 36% sequence similarity. The uORFs conserved between the cereals (rice and at least two others) and Arabidopsis (median value of 36% sequence similarity) generally had a higher amino acid sequence similarity than those uORFs conserved between rice and Arabidopsis (median value of 28% sequence similarity). Given that the uORFs from orthologous genes were selected to be within a given length interval for alignment purposes, the high amino acid sequence similarity may suggest that these uORFs have a functional role (e.g., ribosomal stalling) that is mediated by the encoded uORF peptide.

### Start codon context and codon usage of uORFs

The presence of uORFs does not mean that they will be translated. The sequence context of some plant uORFs has been shown to be sub-optimal for efficient initiation [47,48]. We therefore examined the sequence context of our cereal uORF AUG codons to see if there was any sequence conservation that may aid in their ribosome initiation. We found that there were no informative positions in the uORF consensus sequence context (see Additional file 6 – Tran_FigureS1.eps) based on the observed number of positions that showed sequence conservation was not greater than expected by chance alone. However when the context of the AUGs demarcating the conserved uORFs were compared with the context of the AUG at the main ORF it was evident that the main ORF generally had a better sequence context denoted by a purine in the -3 position and a guanine in the +4 position (Table 5).

**Table 5:** Comparison of conserved cereal uORFs and their main ORF start context'

In five cereals						
Identifier	uORF1	uORF2	uORF3	uORF4	uORF5	Main ORF

AK106095	131_9_17^a ^CCGATGC^b^					157_1179 CCCATGG
AK103391	205_75_74 TTGATGA					354_1116 CAAATGG
AK100589	240_9_334 TGGATGT	248_156_179 CTAATGG	296_108_179 TTGATGT			583_1197 CCAATGG
AK073303	67_9_142 TCCATGC	135_9_74 CTCATGA				218_774 AGCATGG
AK072868	249_27_248 GGAATGC	259_195_70 AAGATGT	269_39_216 TGCATGC	338_90_96 TTCATGA	392_36_96 ACTATGG	524_1332 GTGATGG
AK072649	100_192_117 CTCATGA					409_1443 AAGATGG
AK066145	178_12_58 GCTATGG					248_360 GAGATGG
AK064792	276_15_187 CGGATGC					478_330 GGAATGG
AK060523	173_123_185 ACTATGG					481_501 CGGATGG

In rice and arabidopsis						

Identifier	uORF1	uORF2	uORF3	uORF4	uORF5	Main ORF

AK101100	142_12_21 GCCATGG					175_1578 AAGATGG
AK066952	365_66_182 CCAATGA	368_63_182 ATGATGA	503_51_59 CTGATGA			613_2085 GGGATGC
AK119592	304_90_148 CCGATGA					542_1032 GCGATGG
AK100589	248_156_179 CTAATGG					583_1197 CCAATGG
AK103391	176_30_148 AACATGA	205_75_74 TTGATGA				354_1116 CAAATGG
AK069534	813_9_432 TCGATGA					1254_1602 GAGATGC
AK069526	214_126_544 GATATGG	690_9_185 TTGATGG	820_36_28 CATATGA			884_1455 AAAATGG
AK072868	338_90_96 TTCATGA					524_1332 GTGATGG
AK060523	173_123_185 ACTATGG	206_90_185 CCGATGC				481_501 CGGATGG
AK067412	222_84_49 CTGATGC					355_1059 GGGATGG
AK102277	228_117_150 TCTATGC					495_1017 GAAATGG

^a ^Pre-orf distance_uORF length_intercistronic distance.

^b ^uORF or mainORF sequence context from -3 position to +4.

Recent work showed that ribosome stalling could occur at rare codons [49-53]. We therefore examined the codon usage of the identified uORFs to determine if they contained an increased number of rare codons. We showed that the frequencies of some codons had a *p-Value *less than <*0.05 *in the rice uORF codon usage compared to the rice main coding region based on a significant deviation of observed from expected numbers of uORF codons (Equations 1 and 2); however, the number of codons that had these *p-Values *were not greater than expected by chance (see Additional file 7 – Tran_FigureS2.eps).

## Discussion and conclusion

### Conserved uORFs are rare

This study provides a method to identify conserved uORFs from large assembled EST datasets. We developed a pipeline that used a modified reciprocal best hit method to identify putative orthologous sequences that were then analysed by a comparative R-nomics program called uORFSCAN to find conserved uORFs. We showed that this pipeline was successful in identifying 29 rice uORFs that are conserved at the amino acid level (median value of 36% sequence similarity) in wheat, barley, maize, sorghum, and in some cases (33%) Arabidopsis.

The number of conserved uORFs that share sequence similarity in the transcriptome of cereals appears to be low. This is consistent with reports of conserved uORFs in distantly related plants (i.e., rice and Arabidopsis) [12] and in *Drosophila melanogaster *[14]. One explanation is that genes controlled at the level of translation by uORFs have low levels of transcription [27] and therefore are under- represented in cDNA and assembled EST databases. Another explanation for the low numbers of conserved cereal uORFs is that the uORFs have evolved in both length and sequence such that they no longer share sequence similarity across minor taxonomic groups (i.e., within the cereals) (Table 2 and 4). Furthermore, if the codon usage of cereal uORFs rather than the uORF-encoded peptide were a major controlling mechanism then amino acid sequence may not be conserved.

### Cereal uORFs conserved in Arabidopsis

It has been shown that the amino acid sequence of uORFs in monocot and dicot plants can be similar [46]. Sequence similarity was observed at the amino acid level across the major taxonomic groups (e.g., Arabidopsis and rice) (Table 3). We identified 11 rice genes that contained uORFs conserved in Arabidopsis, of which nine were also conserved in additional cereals (at least two others). For example, a rice cDNA encoding Ankyrin-3 contains an uORF that is conserved in the cereals and Arabidopsis, but it contains a nested uORF that appears to be conserved only in rice and Arabidopsis. Therefore, it is likely that after the split between the two major groups of angiosperms (monocots and dicots), the rice gene has gained an additional in-frame and internal start codon, that is not present in the other cereals, making a nested uORF that is shorter by 33 nucleotides. It would be of interest to determine if the nested uORF is still functional.

Conservation of uORF sequence within the cereals might simply reflect a relatively recent ancestor, rather than conservation of function, therefore it is difficult to predict whether these uORFs are likely to be sequence dependent or sequence independent uORFs [18,19]. However, uORFs that are conserved across both monocots and dicots suggest that these uORFs have a role in a sequence dependent manner. Indeed, six rice uORFs (out of 15, excluding nested uORFs, Table 3) that were conserved in Arabidopsis had a notable amino acid composition that was rich in serine or arginine (at least 20%). It has been suggested that uORF peptides that are rich in serine could either promote or inhibit ribosomal stalling through their phosphorylation [12,54], while arginine rich motifs can be involved in RNA binding [55]. Interestingly, of these six rice uORFs two (AK101100 and AK067412) are found in genes involved in phosphorylation, a function that appears to be over-represented in this dataset (Table 3). We hypothesize that the main protein of these genes could have dual functions, the primary function is as a *trans*-acting factor in an unknown signalling cascade, and a secondary function as a regulator of mORF expression whereby the mORF protein phosphorylates the serine-rich uORF peptides, resulting in a conformational change that allows the uORF peptides to bind and stall ribosomes [16].

There are uORFs previously identified in Arabidopsis that were not identified in this study. For example, the Arabidopsis auxin response factor (ARF) genes [35]*ETTIN (ETT) *and *MONOPTEROS (MP) *contain uORFs and while orthologues of these genes were found in the rice, sorghum and wheat assembled EST datasets, the uORFs showed no sequence similarity (by ClustalW) and were of different lengths (data not shown). Similarly, uORFs found in Arabidopsis genes *AtMHX *and *AtNMT1 *encoding encoding a tonoplast transporter [56] and a phosphoethanolamine N-methyltransferase [57] respectively were not identified because the uORFs were not conserved in rice and at least two other cereals. Finally, the gene containing the uORF in Arabidopsis *sac51 *encoding a bHLH-type transcription factor [58] could not be identified in our rice dataset as we could not identify a clear orthologue. Therefore, it will be of interest to monitor new rice full-length cDNAs and high quality sequences for cereals as they become available to see if more conserved uORFs can be found.

Recently, a pair-wise comparative approach was used to identify conserved uORFs within homology groups that also included paralogs and ohnologs (homologous genes arising by whole-genome duplication) using rice and Arabidopsis full-length cDNAs [12]. Compared to the 11 genes we had identified Hayden and Jorgensen [12] reported that 19 genes contained conserved uORFs between rice and Arabidopsis. Interestingly only four genes (S-Adenosylmethionine decarboxylase, Trehalose-6-phosphate phosphatase, Auxilin-like protein, and Ankyrin-3) were in common highlighting the benefits of complementary search methods. For example, we used the modified reciprocal best hit method to find putative orthologues. It is likely that some of the homologue groups identified by Hayden and Jorgensen [12] may not be true orthologues. For example, homologue group 12 identified by Hayden and Jorgensen [12] were not reciprocal best hit pairs according to our analysis, and therefore are likely to be paralogues. Our approach is deliberately conservative, eliminating paralogues, to maximise the finding of all conserved uORFs independent of their length.

One possible criticism of our approach is that we have included uORFs as short as 9 nt. However, there are two independent reports that showed that the tiny uORF of SAMDC is functional [27,59], although there is controversy regarding the type of effect and conditions under which the tiny uORF of SAMDC exerts its affect on downstream translation. Therefore, there is insufficient data to conclude one way or the other, and as such we have elected to be conservative. This has allowed us to find several genes (e.g. protein phosphatase 2a, a protein containing a ww domain, and GAMYB-binding protein) that were not found by Hayden and Jorgensen's 'uORF-Finder' program [12] because it only detected conserved uORFs greater than 63 codons.

### Better quality assembled EST data is needed

One unavoidable limitation of using incomplete assembled EST data for orthology determination is that orthologues could be falsely assigned in situations where sequences have multiple protein domains. This will increase the number of putative orthologues identified prior to the prediction of uORFs, which is not necessarily harmful as these predictions are manually curated. However, to minimise this problem, we used a sequence coverage cutoff of at least 70% of any of the protein sequences in the alignment (see Methods). We also grouped the orthologues into several datasets representing the number of orthologues that could be found for each gene. For example, the datasets included rice genes that had orthologues in four other cereals (5 out of 5 dataset), in three other cereals (4 out of 5 dataset), and in two other cereals (3 out of 5 dataset). This grouping of orthologues will also help minimise the effects of missing, incomplete, or erroneous assembled EST data.

There are reports of conserved uORFs in monocots and dicots that share high sequence similarity that were not found by our pipeline, due to either lack of sequence conservation or due to limitations in the assembled ESTs currently available. For example, the uORF found in the basic region leucine zipper (bZIP)-type transcription factor *AtB2/AtbZIP11 *was found to be conserved in rice and barley [32], but not in the other cereals included in this study because the sequences are not represented in the other datasets. Current limitations include incomplete data (i.e., not all sequences are represented) and poor quality sequence data, leading to frame-shifts and incorrect prediction of uORFs. Therefore, it is possible to obtain higher numbers of conserved uORFs if the cluster size was relaxed to two out of five, but this approach would reduce the power of comparative R-nomics, and would require significant manual curation.

### Sequence dependent and independent uORF

The cereal uORFs identified here are likely to encode bioactive peptides as selection has occurred at the peptide level. Those cereal uORFs that showed sequence conservation at the amino acid level with Arabidopsis are likely to be classified as sequence-dependent, as the encoded uORF peptide has remained conserved across the angiosperms, suggesting the peptide is directly involved in translational control [9] or has some other biological activity [12-15]. Some identified uORFs were conserved only within the cereals, indicating a relative recent origin or selective loss of the uORFs in Arabidopsis. We cannot rule out the possibility that some conserved cereal uORFs could also act in a sequence-independent manner, as a recent paper reported a conserved uORF in human and mouse ribosomal protein S6 kinase genes (the same finding by our analysis in cereals, Table 1), and suggested that the uORF translational control of the main ORF was through reinitiation [26]. Experiments are needed to confirm the biological activity of the uORF in ribosomal protein S6 kinase gene.

The sequence context surrounding an uORF (ignoring secondary structure) does not appear to play a major role in its recognition and initiation of translation at an uORF. We hypothesize that this sub-optimal uORF sequence context (compared to optimal Kozak consensus [47] sequence for the main coding region) would allow for leaky scanning [48,60] of the uORF, and preferential initiation at the downstream main coding region. An optimal uORF sequence context would provide rigid control in the translational regulation of the main coding region, as initiation would predominantly start at the uORF resulting in reduced availability of initiation factors, such as eIF2, for re-initiation at the downstream main open reading frame.

Sequence-independent uORFs allow for low-level translation of the downstream main coding region [61]. Low-level translation is possible, as sequence-independent uORFs do not cause ribosomal stalling as seen in sequence-dependent uORFs. The regulatory mechanism of the sequence-independent uORF involves other factors (uORF recognition, length, stop codon environment, and the downstream intercistronic sequence) that influence reinitiation efficiency [18,19], and more recently leaky scanning [48], to regulate downstream translation. We analysed the codon usage of conserved uORFs and found no preferential usage of rare codons in the uORFs. Therefore, it is unlikely that the uORF codon usage could contribute to low-level translation as seen for certain rare codons in *Xenopus laevis *[50] and *Eschericia coli *[51] that can reduce translation.

In conclusion, this study showed that the uORFSCAN pipeline is a useful tool for identifying conserved uORFs in closely related species. This pipeline has allowed us to identify 29 conserved uORFs in cereals. Possibly more conserved uORFs will be identified once the cDNA and assembled EST datasets become more comprehensive. These conserved rice uORFs will be useful for future functional analyses that should provide some perspective into downstream translational regulation by uORFs.

## List of abbreviations

5'-UTR: five prime untranslated region; uORF: upstream open reading frame; EST: expressed sequence tag; RBH: reciprocal best hit; CDS: coding sequence.

## Authors' contributions

MT conducted the research, analysed the data and drafted the manuscript. UB and CJS designed the research, participated in the study design, coordinated the study and contributed to the final manuscript. All authors read and approved the final manuscript.

## Supplementary Material

Additional file 1 **TRAN_TableS1**. 'The uORFs predicted by uORFSCAN in 4 out of 5'. Click here for file

Additional file 2 **TRAN_TableS2**. 'The uORFs predicted by uORFSCAN in 3 out of 5' Click here for file

Additional file 3 **TRAN_TableS3**. 'The uORFs predicted by uORFSCAN in 4 out of 4'. Click here for file

Additional file 4 **TRAN_TableS4**. 'The uORFs predicted by uORFSCAN in 3 out of 4'. Click here for file

Additional file 5 **TRAN_TableS5**. 'The uORFs predicted by uORFSCAN in 3 out of 3'. Click here for file

Additional file 6 **Tran_FigureS1**. 'The pattern of nucleotide sequence conservation calculated for the decanucleotide surrounding the uORF AUG triplet using WebLogo [62]'. The overall height of each stack indicates the nucleotide sequence conservation at that position (measured in bits), whereas the height of nucleotide symbols (A, T, G, C) within the stack reflects the relative frequency of the corresponding nucleotide at that position. (B) Positions showing detectable nucleotide sequence conservation were magnified. Click here for file

Additional file 7 **Tran_FigureS2**. 'Relative frequencies of codons showing significant deviation in codon usage between rice uORFs and rice main coding regions'. Rice uORF codon usage calculated from . Click here for file

Additional file 8 **TRAN_TableS6**. 'ClustalW alignment of uORFs identified by uORFSCAN in 5 out of 5 cereals and in Arabidopsis'. Click here for file

Additional file 9 **uORFSCAN**. 'uORFSCAN program'. Click here for file
