# Supplementary material for: Conserved upstream open reading frames in higher plants
Source: BMC Genomics. 2008 Jul 31;9:361. doi: 10.1186/1471-2164-9-361 (PMC2527020; doi:10.1186/1471-2164-9-361)
Supplement: Additional file 1 — TRAN_TableS1. 'The uORFs predicted by uORFSCAN in 4 out of 5'. [file 1471-2164-9-361-S1.doc]

| Table S1. The uORFs predicted by uORFSCAN in 4 out of 5 | | | | | | | | | | | | | | | |
| --- | --- | --- | --- | --- | --- | --- | --- | --- | --- | --- | --- | --- | --- | --- | --- |
| Rice | |  | Wheat | |  | Barley | |  | Maize | |  | Sorghum | | Avg. A.A. similarity (%) | Putative function^b^ |
| Identifier | 5′-UTR^a^ |  | Identifier | 5′-UTR^a^ |  | Identifier | 5′-UTR^a^ |  | Identifier | 5′-UTR^a^ |  | Identifier | 5′-UTR^a^ |
| AK121001 | 90_33_113 |  |  |  |  | TC146266 | 76_33_71 |  | TC279901 | 100_33_67 |  | TC102588 | 118_33_67 | 60 | Transcription factor |
| AK120494 | 199_21_34 |  | TC256417 | 136_21_625 |  | TC134801 | 394_21_404 |  |  |  |  | TC97268 | 166_21_144 | 17 | Hypothetical protein |
| AK119592 | 304_90_148 |  |  |  |  | TC140173 | 311_90_110 |  | TC297985 | 464_90_147 |  | TC103116 | 310_90_302 | 72 | Leucine zipper protein 16 |
|  |  |  |  |  |  |  | 287_144_110 |  |  | 440_114_147 |  |  | 286_114_302 | 68 |  |
|  |  |  |  |  |  |  | 287_144_110 |  |  | 440_114_147 |  |  | 286_114_302 | 70 |  |
| AK104437 | 187_42_203 |  | TC266855 | 203_42_174 |  | TC133317 | 181_42_178 |  | TC282409 | 251_42_164 |  |  |  | 92 | RNA-binding protein cabeza |
| AK103391 | 157_123_74 |  | TC269775 | 203_123_62 |  | TC134190 | 156_123_62 |  | TC294011 | 167_123_75 |  |  |  | 70 | Trehalose |
| AK102376 | 115_24_31 |  | TC237876 | 95_24_48 |  | TC133824 | 87_24_47 |  |  |  |  | TC101133 | 576_24_21 | 13 | Zinc finger protein-like |
| AK102277 | 267_78_150 |  | TC250018 | 255_78_130 |  |  |  |  | TC299034 | 266_78_144 |  | TC102365 | 258_78_137 | 92 | AP2 domain-containing protein |
|  | 228_117_150 |  |  | 216_117_130 |  |  |  |  |  | 227_117_144 |  |  | 219_117_137 | 82 |  |
|  | 126_219_150 |  |  | 108_225_130 |  |  |  |  |  | 131_213_144 |  |  | 120_216_137 | 65 |  |
| AK102068 | 463_12_11 |  | TC243607 | 181_12_14 |  | TC136167 | 397_12_315 |  |  |  |  | TC103028 | 402_12_14 | 33 |  |
| AK100578 | 249_9_10 |  | TC241920 | 568_9_372 |  |  |  |  | TC300179 | 180_9_136 |  | TC103751 | 83_9_62 | 50 | mRNA capping enzyme-like |
| AK099540 | 277_6_475 |  |  |  |  | TC139607 | 184_6_495 |  | TC280858 | 23_6_227 |  | TC101936 | 513_6_465 | 100 | Nam-like protein 2 |
| AK073985 | 101_12_101 |  | TC252583 | 179_12_900 |  | TC148772 | 149_12_82 |  |  |  |  | TC92492 | 123_12_329 | 33 | RNA-binding protein FUS |
| AK070766 | 144_15_65 |  | TC263230 | 129_15_44 |  | TC134132 | 121_15_44 |  | TC305003 | 186_15_43 |  |  |  | 50 | PG4 |
| AK065585 | 126_15_34 |  | TC254095 | 64_15_42 |  | TC139863 | 34_15_48 |  | TC311554 | 192_15_22 |  |  |  | 100 | Monodehydroascorbate |
| AK065240 | 112_21_17 |  |  |  |  | TC132139 | 231_21_13 |  | TC298549 | 354_21_28 |  | TC93046 | 63_21_111 | 83 | Arabinofuranohydrolase |
| AK065176 | 333_12_179 |  | TC235016 | 413_12_202 |  | TC139184 | 466_12_203 |  |  |  |  | TC93049 | 357_12_192 | 67 | Phosphatidylinositol |
| AK061109 | 35_12_98 |  | TC263378 | 53_12_68 |  |  |  |  | TC306071 | 79_12_66 |  | TC96170 | 81_12_57 | 33 | Hypothetical protein |
| ^a^ Pre orf distance_uORF length_intercistronic distance.  ^b^ Functional annotation based on “The UniProt Knowledgebase (UniProt)” database.  Identifiers may not be unique among the tables as different combinations of uORFs were conserved.  Ribosomal rRNA genes have been removed. | | | | | | | | | | | | | | | |
