# Supplementary material for: Conserved upstream open reading frames in higher plants
Source: BMC Genomics. 2008 Jul 31;9:361. doi: 10.1186/1471-2164-9-361 (PMC2527020; doi:10.1186/1471-2164-9-361)
Supplement: Additional file 2 — TRAN_TableS2. 'The uORFs predicted by uORFSCAN in 3 out of 5' [file 1471-2164-9-361-S2.doc]

| Table S2. The uORFs predicted by uORFSCAN in 3 out of 5 | | | | | | | | | | | | | | | |
| --- | --- | --- | --- | --- | --- | --- | --- | --- | --- | --- | --- | --- | --- | --- | --- |
| Rice | |  | Wheat | |  | Barley | |  | Maize | |  | Sorghum | | Avg. A.A. similarity (%) | Putative function^b^ |
| Identifier | 5′-UTR^a^ |  | Identifier | 5′-UTR^a^ |  | Identifier | 5′-UTR^a^ |  | Identifier | 5′-UTR^a^ |  | Identifier | 5′-UTR^a^ |
| AK122113 | 73_12_635 |  |  |  |  |  |  |  | TC283896 | 249_12_198 |  | TC99912 | 357_12_273 | 33 | Unknown protein |
| AK121846 | 204_39_102 |  |  |  |  |  |  |  | TC292285 | 37_39_477 |  | TC103358 | 136_39_228 | 25 | K+ efflux antiporter |
| AK121416 | 625_36_180 |  |  |  |  | TC134160 | 116_36_41 |  | TC282124 | 164_36_38 |  |  |  | 1 | DNA-directed RNA polymerase II |
|  | 571_33_237 |  |  |  |  |  | 119_33_41 |  |  |  |  | TC105440 | 34_33_140 | 10 |  |
|  | 251_126_464 |  |  |  |  |  |  |  |  | 102_126_10 |  |  | 38_132_37 | 2 |  |
|  | 248_129_464 |  |  |  |  |  |  |  |  | 102_126_10 |  |  | 38_132_37 | 2 |  |
| AK121398 | 120_27_63 |  |  |  |  |  |  |  | TC311365 | 164_27_38 |  | TC104131 | 111_27_82 | 40 | Hypothetical protein |
| AK121128 | 1194_63_16 |  |  |  |  |  |  |  | TC297930 | 132_63_301 |  | TC106329 | 387_66_345 | 5 | AC transposase |
|  | 1011_60_202 |  |  |  |  |  |  |  | TC297930 | 132_63_301 |  |  | 642_57_99 | 1 |  |
| AK120409 | 257_12_1201 |  | TC253102 | 218_12_752 |  |  |  |  | TC313498 | 271_12_255 |  |  |  | 100 | Cyclin T1 |
| AK120173 | 386_51_59 |  | TC252694 | 391_51_358 |  |  |  |  |  |  |  | TC93304 | 397_51_258 | 1 | Hypothetical protein |
|  | 33_30_433 |  |  | 36_30_734 |  |  |  |  |  |  |  |  | 183_30_493 | 56 |  |
| AK111899 | 168_21_68 |  | TC254194 | 18_21_260 |  |  |  |  |  |  |  | TC105689 | 513_21_42 | 1 | Response regulator 10 |
| AK111821 | 978_69_878 |  | TC269697 | 396_66_78 |  |  |  |  |  |  |  | TC96287 | 342_66_372 | 9 | Transcription factor MYB86 |
|  | 795_75_1055 |  |  | 390_72_78 |  |  |  |  |  |  |  |  | 336_72_372 | 4 |  |
|  | 212_69_1644 |  |  | 390_72_78 |  |  |  |  |  |  |  |  | 336_72_372 | 8 |  |
|  | 1223_63_639 |  |  | 396_66_78 |  |  |  |  |  |  |  |  | 342_66_372 | 10 |  |
|  | 1125_15_785 |  |  | 360_15_165 |  |  |  |  |  |  |  |  | 306_15_459 | 50 |  |
| AK111748 | 540_12_55 |  |  |  |  | TC142603 | 21_12_95 |  | TC300604 | 546_12_29 |  |  |  | 33 | Ethylene receptor-like protein 1 |
| AK105484 | 181_96_51 |  | TC235660 | 198_93_56 |  |  |  |  |  |  |  | TC93642 | 173_99_47 | 17 | Homeodomain leucine zipper protein |
| AK103391 | 176_30_148 |  | TC269775 | 222_30_136 |  |  |  |  | TC294011 | 186_30_149 |  |  |  | 56 | Trehalose-6-phosphate phosphatase |
|  | 130_27_197 |  |  | 176_27_185 |  | TC134190 | 129_27_185 |  |  |  |  |  |  | 38 |  |
|  | 118_39_197 |  |  | 164_39_185 |  | TC134190 | 117_39_185 |  |  |  |  |  |  | 50 |  |
| AK102966 | 206_9_32 |  | TC247483 | 188_9_14 |  | TC142783 | 160_9_138 |  |  |  |  |  |  | 50 | Type 5 serine/threonine phosphatase |
| AK102080 | 899_9_127 |  |  |  |  |  |  |  | TC298645 | 131_9_125 |  | TC107852 | 461_9_24 | 50 | Arm repeat-containing protein |
|  | 491_9_535 |  |  |  |  |  |  |  |  | 131_9_125 |  |  | 461_9_24 | 50 |  |
|  | 272_6_757 |  |  |  |  |  |  |  |  | 123_6_136 |  |  | 33_6_455 | 100 |  |
| AK101720 | 152_9_74 |  | TC270620 | 187_9_264 |  |  |  |  | TC289352 | 188_9_675 |  |  |  | 50 | Probable calcium-binding |
| AK101319 | 976_9_280 |  |  |  |  | TC142174 | 177_9_335 |  | TC298112 | 153_9_176 |  |  |  | 50 | Hypothetical protein |
|  | 898_72_295 |  |  |  |  |  | 446_75_0 |  |  | 75_72_191 |  |  |  | 12 |  |
|  | 544_75_646 |  |  |  |  |  | 446_75_0 |  |  | 75_72_191 |  |  |  | 16 |  |
|  | 532_87_646 |  | TC271530 | 20_87_40 |  |  | 434_87_0 |  |  |  |  |  |  | 14 |  |
|  | 490_129_646 |  |  |  |  |  | 392_129_0 |  |  | 136_123_79 |  |  |  | 10 |  |
|  | 269_9_987 |  |  |  |  |  | 177_9_335 |  |  | 153_9_176 |  |  |  | 50 |  |
| AK101100 | 142_12_21 |  | TC263224 | 132_12_14 |  | TC132639 | 175_12_510 |  |  |  |  |  |  | 100 | Protein phosphatase 2A 55 kDa |
| AK100780 | 276_21_83 |  |  |  |  | TC141120 | 409_21_258 |  |  |  |  | TC105228 | 86_21_152 | 14 | Activin receptor type II precursor |
| AK100589 | 300_54_229 |  |  |  |  |  |  |  | TC292591 | 343_54_218 |  | TC91317 | 317_54_227 | 94 | S-adenosylmethionine decarboxylase |
| AK100299 | 692_21_187 |  | TC239370 | 264_21_11 |  |  |  |  |  | 574_21_163 |  |  |  | 1 |  |
| AK099745 | 136_21_245 |  | TC269480 | 129_21_140 |  | TC136177 | 22_21_245 |  |  |  |  |  |  | 17 | Glutamate receptor 3.2 precursor |
| AK099540 | 145_90_523 |  |  |  |  | TC13960 | 7 52_90_543 |  |  |  |  | TC101936 | 381_90_513 | 83 | Nam-like protein 2 |
| AK072868 | 377_51_96 |  | TC247418 | 389_51_111 |  | TC139536 | 429_51_117 |  |  |  |  |  |  | 81 | Serine/threonine kinase |
| AK072499 | 555_90_1239 |  |  |  |  | TC139601 | 252_87_88 |  |  |  |  | TC105154 | 157_93_526 | 10 | Short stature homeobox protein 2 |
|  | 416_9_1459 |  | TC267242 | 82_9_80 |  |  |  |  |  |  |  |  | 431_9_336 | 50 |  |
|  | 317_69_1498 |  |  | 62_72_37 |  |  |  |  | TC281509 | 24_66_263 |  |  |  | 1 |  |
|  | 210_33_1641 |  |  |  |  |  | 63_33_331 |  |  |  |  |  | 485_33_258 | 10 |  |
|  | 1675_66_143 |  |  |  |  |  |  |  |  | 24_66_263 |  |  | 455_63_258 | 9 |  |
|  | 1391_96_397 |  |  |  |  |  |  |  |  | 90_96_167 |  |  | 157_93_526 | 1 |  |
|  | 1258_66_560 |  |  |  |  |  |  |  |  | 24_66_263 |  |  | 455_63_258 | 1 |  |
|  | 1159_9_716 |  |  | 82_9_80 |  |  |  |  |  |  |  |  | 740_9_27 | 50 |  |
|  | 1094_9_781 |  |  | 82_9_80 |  |  |  |  |  |  |  |  | 740_9_27 | 50 |  |
| AK072427 | 7_27_136 |  | TC258198 | 99_27_41 |  |  |  |  | TC308361 | 214_27_640 |  |  |  | 13 | Hypothetical protein |
| AK072349 | 376_9_36 |  |  |  |  | TC137384 | 305_9_235 |  | TC313267 | 310_9_31 |  |  |  | 100 | Enhancer of polycomb-like protein |
| AK071762 | 87_12_116 |  |  |  |  | TC131045 | 140_12_347 |  |  |  |  | TC101994 | 100_12_109 | 33 | Wali7 protein |
| AK070751 | 664_33_209 |  | TC240522 | 226_33_83 |  | TC142763 | 298_33_5 |  |  |  |  |  |  | 9 | F7N22.3 protein |
|  | 501_60_345 |  |  |  |  |  | 208_60_68 |  |  |  |  | TC106350 | 122_57_42 | 1 |  |
|  | 398_6_502 |  | TC240522 | 264_6_72 |  |  |  |  | TC294109 | 238_6_201 |  |  |  | 100 |  |
| AK070456 | 774_9_51 |  |  |  |  |  |  |  | TC288447 | 587_9_472 |  | TC97361 | 72_9_118 | 50 | Molybdenum cofactor Cnx1 |
| AK069730 | 770_156_22 |  | TC246998 | 270_150_246 |  | TC132118 | 275_159_249 |  |  |  |  |  |  | 15 | Unknown protein |
|  | 412_153_383 |  |  | 270_150_246 |  |  | 275_159_249 |  |  |  |  |  |  | 47 |  |
| AK067468 | 3_6_164 |  |  |  |  | TC138312 | 401_6_126 |  | TC294470 | 533_6_178 |  |  |  | 100 | Phosphatidylinositol 3,5-kinase-like |
| AK066942 | 259_12_32 |  | TC253984 | 286_12_43 |  | TC133589 | 262_12_36 |  |  |  |  |  |  | 67 | Expressed protein |
| AK066073 | 154_75_125 |  | TC236575 | 204_75_379 |  |  |  |  | TC293675 | 596_75_169 |  |  |  | 1 | Acetyl-coenzyme A synthetase |
| AK065538 | 162_24_57 |  |  |  |  | TC139620 | 197_24_39 |  | TC287533 | 93_24_70 |  |  |  | 14 | Clathrin coat assembly protein |
| AK065237 | 62_9_226 |  |  |  |  |  |  |  | TC288346 | 32_9_124 |  | TC10281 | 0 65_9_245 | 50 | Expressed protein |
|  | 174_9_114 |  |  |  |  |  |  |  |  | 152_9_4 |  | TC102810 | 65_9_245 | 50 |  |
| AK065176 | 315_30_179 |  | TC235016 | 395_30_202 |  | TC139184 | 448_30_203 |  |  |  |  |  |  | 44 | Phosphatidylinositol 3-and 4-kinase-like |
| AK065137 | 8_21_281 |  | TC251833 | 13_21_266 |  | TC147261 | 7_21_263 |  |  |  |  |  |  | 83 | Kelch-like ECH-associated protein 1 |
| AK064792 | 281_99_98 |  | TC267323 | 259_99_98 |  |  |  |  | TC306152 | 268_99_98 |  |  |  | 72 | Hypothetical protein |
|  | 276_15_187 |  |  | 254_15_187 |  |  |  |  |  | 263_15_187 |  |  |  | 100 |  |
| AK061004 | 108_9_30 |  | TC269443 | 96_9_25 |  | TC151138 | 130_9_25 |  |  |  |  |  |  | 100 | Peptidylprolyl isomerase |
| AK060780 | 546_6_320 |  |  |  |  | TC134531 | 513_6_122 |  | TC311790 | 53_6_34 |  |  |  | 100 | Pelota (PEL1) |
|  | 440_6_426 |  |  |  |  |  | 513_6_122 |  |  | 53_6_34 |  |  |  | 100 |  |
| AK060523 | 60_27_394 |  |  |  |  |  |  |  | TC305149 | 127_27_425 |  | TC103609 | 133_27_421 | 75 | Ankyrin-2 |
| AK058965 | 4_186_71 |  |  |  |  |  |  |  | TC288549 | 102_186_226 |  | TC93810 | 51_186_67 | 75 | Nitrilase 1 |
| AK058513 | 94_24_26 |  |  |  |  | TC147191 | 616_24_26 |  | TC305089 | 645_24_26 |  |  |  | 57 | Leucine aminopeptidase pre protein |
|  | 34_84_26 |  |  |  |  |  | 556_84_26 |  |  | 585_84_26 |  |  |  | 63 |  |
|  | 128_6_10 |  |  |  |  |  | 640_6_20 |  |  | 669_6_20 |  |  |  | 100 |  |
|  | 118_24_2 |  |  |  |  |  | 616_24_26 |  |  | 645_24_26 |  |  |  | 14 |  |
| AK058988 | 139_69_294 |  | TC235910 | 265_69_267 |  |  |  |  | TC314670 | 272_69_84 |  |  |  | 78 | Calcium-binding protein like |
| ^a^ Pre orf distance_uORF length_intercistronic distance  ^b^ Functional annotation based on “The UniProt Knowledgebase (UniProt)” database  Identifiers may not be unique among the tables as different combinations of uORFs were conserved.  Ribosomal rRNA genes have been removed. | | | | | | | | | | | | | | | |
