# Supplementary material for: Conserved upstream open reading frames in higher plants
Source: BMC Genomics. 2008 Jul 31;9:361. doi: 10.1186/1471-2164-9-361 (PMC2527020; doi:10.1186/1471-2164-9-361)
Supplement: Additional file 3 — TRAN_TableS3. 'The uORFs predicted by uORFSCAN in 4 out of 4'. [file 1471-2164-9-361-S3.doc]

| Table S3. The uORFs predicted by uORFSCAN in 4 out of 4 | | | | | | | | | | | | |
| --- | --- | --- | --- | --- | --- | --- | --- | --- | --- | --- | --- | --- |
| Rice | |  | Wheat | |  | Barley | |  | Maize | | Avg. A.A. similarity (%) | Putative function^b^ |
| Identifer | 5′-UTR |  | Identifer | 5′-UTR^a^ |  | Identifer | 5′-UTR^a^ |  | Identifer | 5′-UTR^a^ |
| AK121850 | 86_18_51 |  | TC238796 | 102_18_57 |  | TC140406 | 84_18_58 |  | TC292944 | 94_18_72 | 20 | Protein kinase CK2 |
| AK104437 | 187_42_203 |  | TC266855 | 203_42_174 |  | TC133317 | 181_42_178 |  | TC282409 | 251_42_164 | 92 | RNA-binding protein cabeza |
| AK103140 | 271_36_1 |  | TC266113 | 212_36_407 |  | TC140479 | 182_36_1 |  | TC281091 | 194_36_1 | 64 | Protein phosphatase 2C |
| AK101684 | 158_21_12 |  | TC253407 | 137_21_33 |  | TC141318 | 104_21_33 |  | TC307223 | 180_21_16 | 33 | CCAAT-binding transcription factor |
| AK100440 | 246_81_195 |  | TC235293 | 210_78_152 |  | TC133630 | 180_78_153 |  | TC280879 | 138_78_77 | 4 | BZIP transcription factor |
| AK099839 | 147_48_82 |  | TC237323 | 145_48_50 |  | TC140250 | 758_48_585 |  | TC309986 | 144_48_57 | 7 | MAP3K epsilon protein kinase |
| AK073303 | 67_9_142 |  | TC237149 | 75_9_113 |  | TC132556 | 81_9_139 |  | TC305609 | 127_9_69 | 50 | Hypothetical protein |
|  | 135_9_74 |  |  | 75_9_113 |  |  | 81_9_139 |  |  | 127_9_69 | 50 |  |
| AK072868 | 392_36_96 |  | TC247418 | 404_36_111 |  | TC139536 | 444_36_117 |  | TC306591 | 633_36_366 | 8 | Serine/threonine kinase |
|  | 338_90_96 |  |  | 347_93_111 |  |  | 387_93_117 |  |  | 576_93_366 | 6 |  |
|  | 269_39_216 |  |  | 278_39_234 |  |  | 318_39_240 |  |  | 768_39_228 | 8 |  |
|  | 259_195_70 |  |  | 268_198_85 |  |  | 308_198_91 |  |  | 260_192_583 | 35 |  |
|  | 249_27_248 |  |  | 258_27_266 |  |  | 298_27_272 |  |  | 444_27_564 | 11 |  |
| AK072649 | 100_192_117 |  | TC236348 | 79_192_117 |  | TC133316 | 76_192_93 |  | TC305793 | 180_192_116 | 81 | Ribosomal protein S6 kinase homolog |
| AK070766 | 144_15_65 |  | TC263230 | 129_15_44 |  | TC134132 | 121_15_44 |  | TC305003 | 186_15_43 | 50 | Protein C20orf11 |
| AK069526 | 737_87_60 |  | TC265553 | 757_87_62 |  | TC147034 | 740_87_62 |  | TC287352 | 477_87_65 | 32 | GAMYB-binding protein |
|  | 690_9_185 |  |  | 709_9_188 |  |  | 692_9_188 |  |  | 427_9_193 | 50 |  |
|  | 440_102_342 |  |  | 453_102_351 |  |  | 436_102_351 |  |  | 150_102_377 | 12 |  |
| AK066145 | 178_12_58 |  | TC266262 | 149_12_73 |  | TC134484 | 154_12_231 |  | TC286452 | 224_12_70 | 33 | F2E2.12 |
| AK065585 | 126_15_34 |  | TC254095 | 64_15_42 |  | TC139863 | 34_15_48 |  | TC311554 | 192_15_22 | 100 | Monodehydroascorbate reductase |
| AK063875 | 128_78_44 |  | TC238591 | 348_78_2 |  | TC133365 | 519_78_2 |  | TC312696 | 325_78_2 | 12 | Prokineticin 2 precursor |
| AK060523 | 173_123_185 |  | TC235416 | 201_126_157 |  | TC148319 | 211_120_163 |  | TC305149 | 255_129_195 | 60 | Ankyrin-3 |
| AK060232 | 38_15_10 |  | TC273755 | 224_15_44 |  | TC135479 | 410_15_99 |  | TC291309 | 171_15_4 | 25 | SAM-dependent methyltransferase-like |
| ^a^ Pre orf distance_uORF length_intercistronic distance  ^b^ Functional annotation based on “The UniProt Knowledgebase (UniProt)” database  Identifiers may not be unique among the tables as different combinations of uORFs were conserved.  Ribosomal rRNA genes have been removed. | | | | | | | | | | | | |
