# Supplementary material for: Conserved upstream open reading frames in higher plants
Source: BMC Genomics. 2008 Jul 31;9:361. doi: 10.1186/1471-2164-9-361 (PMC2527020; doi:10.1186/1471-2164-9-361)
Supplement: Additional file 4 — TRAN_TableS4. 'The uORFs predicted by uORFSCAN in 3 out of 4'. [file 1471-2164-9-361-S4.doc]

| Table S4. The uORFs predicted by uORFSCAN in 3 out of 4 | | | | | | | | | | | | |
| --- | --- | --- | --- | --- | --- | --- | --- | --- | --- | --- | --- | --- |
| Rice | |  | Wheat | |  | Barley | |  | Maize | | Avg. A.A. similarity (%) | Putative function^b^ |
| Identifier | 5′-UTR |  | Identifier | 5′-UTR^a^ |  | Identifier | 5′-UTR^a^ |  | Identifier | 5′-UTR^a^ |
| AK121416 | 625_36_180 |  |  |  |  | TC134160 | 116_36_41 |  | TC282124 | 164_36_38 | 1 | DNA-directed RNA polymerase |
| AK121122 | 743_21_34 |  | TC266483 | 118_21_70 |  | TC148472 | 179_21_69 |  |  |  | 14 | SNF protein |
| AK121001 | 90_33_113 |  |  |  |  | TC146266 | 76_33_71 |  | TC279901 | 100_33_67 | 60 | Transcription factor |
| AK120494 | 199_21_34 |  | TC256417 | 136_21_625 |  | TC134801 | 394_21_404 |  |  |  | 17 | Hypothetical protein |
| AK120409 | 257_12_1201 |  | TC253102 | 218_12_752 |  |  |  |  | TC313498 | 271_12_255 | 100 | Cyclin T1 |
| AK119650 | 98_21_61 |  | TC247011 | 129_21_73 |  | TC148824 | 219_21_8 |  |  |  | 17 | MAP kinase MAPK2 |
| AK119592 | 304_90_148 |  |  |  |  | TC140173 | 311_90_110 |  | TC297985 | 464_90_147 | 72 | Homeodomain leucine zipper protein 16 |
|  | 283_111_148 |  |  |  |  |  | 287_114_110 |  |  | 440_114_147 | 68 |  |
|  | 280_114_148 |  |  |  |  |  | 287_114_110 |  |  | 440_114_147 | 70 |  |
| AK111887 | 244_21_108 |  | TC235829 | 289_21_48 |  | TC131138 | 232_21_48 |  |  |  | 67 | Calcineurin B protein |
| AK111883 | 623_12_193 |  | TC232511 | 688_12_250 |  |  |  |  | TC295627 | 422_12_387 | 25 | Hypothetical WD-repeat protein |
|  | 508_45_275 |  |  | 24_45_881 |  |  |  |  |  | 497_45_279 | 64 |  |
|  | 433_54_341 |  |  | 195_54_701 |  |  |  |  |  | 236_54_531 | 6 |  |
|  | 348_12_468 |  |  | 139_12_799 |  |  |  |  |  | 338_12_471 | 33 |  |
|  | 300_60_468 |  |  | 288_60_602 |  |  |  |  |  | 290_60_471 | 15 |  |
|  | 246_54_528 |  |  | 195_54_701 |  |  |  |  |  | 236_54_531 | 6 |  |
| AK111748 | 540_12_55 |  |  |  |  | TC142603 | 21_12_95 |  | TC300604 | 546_12_29 | 33 | Ethylene receptor-like protein 1 |
| AK111699 | 401_9_0 |  | TC258667 | 378_9_360 |  |  |  |  | TC307333 | 371_9_0 | 100 | tRNA-dihydrouridine synthase 3 |
| AK106310 | 547_72_149 |  | TC273695 | 366_72_174 |  | TC136869 | 1_72_168 |  |  |  | 21 | Hypothetical protein |
|  | 128_21_619 |  |  | 471_21_120 |  |  | 106_21_114 |  |  |  | 17 |  |
| AK103631 | 376_15_48 |  |  |  |  | TC139875 | 822_15_0 |  | TC306875 | 445_15_0 | 25 | Hypothetical protein |
|  | 328_24_87 |  | TC253392 | 188_24_308 |  |  | 224_24_589 |  |  |  | 13 |  |
|  | 313_39_87 |  |  | 173_39_308 |  |  | 209_39_589 |  |  |  | 17 |  |
|  | 277_18_144 |  |  |  |  |  | 3_18_816 |  | TC306875 | 442_18_0 | 20 |  |
| AK103390 | 277_51_25 |  | TC236507 | 242_51_24 |  | TC132330 | 379_51_24 |  |  |  | 69 | Hypothetical protein |
| AK103207 | 167_9_20 |  | TC269820 | 173_9_475 |  |  |  |  | TC306369 | 149_9_25 | 50 | protein kinase |
| AK103040 | 8_54_271 |  | TC254504 | 13_54_233 |  |  |  |  | TC293848 | 19_54_248 | 35 | Single myb histone 1 |
|  | 45_87_201 |  | TC254504 |  |  | TC148672 | 25_84_166 |  |  |  | 64 |  |
| AK102966 | 206_9_32 |  | TC247483 | 188_9_14 |  | TC142783 | 160_9_138 |  |  |  | 50 | Type 5 serine/threonine phosphatase 55 |
| AK102376 | 115_24_31 |  | TC237876 | 95_24_48 |  | TC133824 | 87_24_47 |  |  |  | 14 | Zinc finger (C3HC4-type RING finger) |
| AK102370 | 127_60_11 |  | TC255624 | 139_63_11 |  | TC133336 | 401_57_20 |  |  |  | 30 | Tubby-like protein 3 |
|  | 124_63_11 |  |  | 139_63_11 |  |  |  |  | TC312575 | 64_66_92 | 10 |  |
| AK102277 | 267_78_150 |  | TC250018 | 255_78_130 |  |  |  |  | TC299034 | 266_78_144 | 92 | Unknown protein |
|  | 228_117_150 |  |  | 216_117_130 |  |  |  |  |  | 227_117_144 | 82 |  |
|  | 126_219_150 |  |  | 108_225_130 |  |  |  |  |  | 131_213_144 | 65 |  |
| AK102068 | 463_12_11 |  | TC243607 | 181_12_14 |  | TC136167 | 397_12_315 |  |  |  | 33 | Hypothetical protein |
| AK101942 | 106_18_51 |  | TC248321 | 82_18_27 |  |  |  |  | TC310601 | 4_18_283 | 40 | Calcium-dependent protein kinase |
| AK101720 | 152_9_74 |  | TC270620 | 187_9_264 |  |  |  |  | TC289352 | 188_9_675 | 50 | Probable calcium-binding mitochondrial |
| AK101520 | 222_30_1281 |  |  |  |  | TC136686 | 134_30_360 |  | TC297598 | 227_30_3 | 11 | protein F19P19.26 |
| AK101319 | 976_9_280 |  |  |  |  | TC142174 | 177_9_335 |  | TC298112 | 153_9_176 | 50 | Hypothetical protein |
|  | 898_72_295 |  |  |  |  |  | 446_75_0 |  |  | 75_72_191 | 12 |  |
|  | 544_75_646 |  |  |  |  |  | 446_75_0 |  |  | 75_72_191 | 16 |  |
|  | 532_87_646 |  | TC271530 | 20_87_40 |  |  | 434_87_0 |  |  |  | 14 |  |
|  | 490_129_646 |  |  |  |  |  | 392_129_0 |  |  | 136_123_79 | 10 |  |
|  | 269_9_987 |  |  |  |  |  | 177_9_335 |  |  | 153_9_176 | 50 |  |
| AK101266 | 493_9_69 |  |  |  |  | TC130775 | 526_9_432 |  | TC285008 | 333_9_90 | 50 | Thiol protease aleurain precursor |
|  | 464_9_98 |  |  |  |  |  | 526_9_432 |  |  | 333_9_90 | 50 |  |
|  | 457_45_69 |  |  |  |  |  | 301_45_621 |  |  | 270_45_117 | 14 |  |
| AK101100 | 142_12_21 |  | TC263224 | 132_12_14 |  | TC132639 | 175_12_510 |  |  |  | 100 | Protein phosphatase 2A 55 kDa B |
| AK100578 | 249_9_10 |  | TC241920 | 568_9_372 |  |  |  |  | TC300179 | 180_9_136 | 50 | MRNA capping enzyme-like protein |
| AK100539 | 301_45_11 |  | TC236703 | 339_45_11 |  |  |  |  | TC305240 | 183_45_11 | 93 | Dentin sialophosphoprotein 1 |
| AK100332 | 50_9_2019 |  | TC272800 | 48_9_152 |  | TC153584 | 275_9_342 |  |  |  | 50 | Chromodomain helicase DNA binding |
|  | 262_78_1738 |  |  | 69_75_65 |  |  | 71_81_474 |  |  |  | 4 |  |
|  | 1769_9_300 |  |  | 48_9_152 |  |  | 275_9_342 |  |  |  | 50 |  |
|  | 1744_87_247 |  |  | 57_87_65 |  |  | 479_90_57 |  |  |  | 3 |  |
|  | 1657_9_412 |  |  | 48_9_152 |  |  | 275_9_342 |  |  |  | 50 |  |
|  | 1513_78_487 |  |  | 69_75_65 |  |  | 71_81_474 |  |  |  | 7 |  |
|  | 1505_9_564 |  |  | 48_9_152 |  |  | 275_9_342 |  |  |  | 50 |  |
|  | 1435_9_634 |  |  | 48_9_152 |  |  | 275_9_342 |  |  |  | 50 |  |
|  | 134_72_1872 |  |  | 72_72_65 |  |  | 80_72_474 |  |  |  | 1 |  |
| AK100299 | 692_21_187 |  | TC239370 | 264_21_11 |  |  |  |  | TC291351 | 574_21_163 | 1 | Hypothetical protein |
| AK100037 | 449_33_85 |  | TC234512 | 400_33_495 |  | TC134276 | 222_33_507 |  |  |  | 90 | SAC domain-containing protein |
| AK099852 | 906_9_2 |  | TC233509 | 103_9_218 |  | TC144509 | 159_9_177 |  |  |  | 50 | Hypothetical protein |
| AK099745 | 136_21_245 |  | TC269480 | 129_21_140 |  | TC136177 | 22_21_245 |  |  |  | 17 | Glutamate receptor 3.2 |
| AK099676 | 77_18_8 |  | TC247479 | 425_18_669 |  |  |  |  | TC294642 | 38_18_8 | 20 | ATPase |
| AK099625 | 353_9_26 |  |  |  |  | TC140108 | 238_9_26 |  | TC281333 | 102_9_25 | 100 | Hypothetical protein |
| AK099540 | 277_6_475 |  |  |  |  | TC139607 | 184_6_495 |  | TC280858 | 23_6_227 | 100 | Nam-like protein 2 |
| AK074023 | 6_75_94 |  |  |  |  | TC145114 | 158_78_142 |  | TC306013 | 274_72_248 | 4 | Hypothetical protein |
| AK073985 | 101_12_101 |  | TC252583 | 179_12_900 |  | TC148772 | 149_12_82 |  |  |  | 67 | RNA-binding protein FUS |
| AK072868 | 377_51_96 |  | TC247418 | 389_51_111 |  | TC139536 | 429_51_117 |  |  |  | 81 | Serine/threonine kinase |
| AK072769 | 272_156_77 |  | TC265505 | 208_150_83 |  |  |  |  | TC292123 | 215_153_83 | 20 | Hypothetical protein |
| AK072499 | 317_69_1498 |  | TC267242 | 62_72_37 |  |  |  |  | TC281509 | 24_66_263 | 1 | Short stature homeobox |
| AK072427 | 7_27_136 |  | TC258198 | 99_27_41 |  |  |  |  | TC308361 | 214_27_640 | 13 | Hypothetical protein |
| AK072349 | 376_9_36 |  |  |  |  | TC137384 | 305_9_235 |  | TC313267 | 310_9_31 | 100 | Enhancer of polycomb-like protein, |
| AK070751 | 664_33_209 |  | TC240522 | 226_33_83 |  | TC142763 | 298_33_5 |  |  |  | 9 | F7N22.3 protein |
|  | 398_6_502 |  |  | 264_6_72 |  |  |  |  | TC294109 | 238_6_201 | 100 |  |
| AK069730 | 770_156_22 |  | TC246998 | 270_150_246 |  | TC132118 | 275_159_249 |  |  |  | 15 | Hypothetical protein |
|  | 412_153_383 |  |  | 270_150_246 |  |  | 275_159_249 |  |  |  | 47 |  |
| AK069726 | 120_78_82 |  | TC235568 | 119_78_74 |  | TC139583 | 107_78_72 |  |  |  | 80 | CBL-interacting protein kinase 23 |
| AK069526 | 214_126_544 |  | TC265553 | 239_123_544 |  | TC147034 | 222_123_544 |  |  |  | 80 | GAMYB-binding protein |
|  | 149_246_489 |  |  | 174_243_489 |  |  | 157_243_489 |  |  |  | 63 |  |
| AK069065 | 133_12_97 |  | TC266624 | 198_12_77 |  | TC132959 | 163_12_73 |  |  |  | 33 | RAD23-like protein |
| AK068416 | 254_33_43 |  | TC239989 | 34_33_29 |  |  |  |  | TC287928 | 312_33_796 | 20 | Expressed protein |
| AK067468 | 3_6_164 |  |  |  |  | TC138312 | 401_6_126 |  | TC294470 | 533_6_178 | 100 | Phosphatidylinositol 3,5-kinase-like |
| AK067412 | 222_84_49 |  | TC252944 | 247_81_102 |  | TC142664 | 123_84_118 |  |  |  | 19 | Protein kinase |
| AK067258 | 246_27_25 |  | TC247646 | 508_27_43 |  | TC140304 | 193_27_46 |  |  |  | 38 | Ankyrin-like protein |
| AK067123 | 840_72_630 |  |  |  |  | TC132179 | 380_75_759 |  | TC300140 | 66_69_62 | 4 | Ubiquitin-specific protease 12 |
|  | 579_69_894 |  |  |  |  |  | 963_66_185 |  |  | 66_69_62 | 1 |  |
|  | 291_72_1179 |  |  |  |  |  | 380_75_759 |  |  | 66_69_62 | 4 |  |
| AK066952 | 437_57_119 |  | TC271435 | 169_57_23 |  | TC137456 | 357_57_231 |  |  |  | 9 | Arabidopsis thaliana genomic DNA |
|  | 392_39_182 |  |  | 153_39_57 |  |  | 222_39_384 |  |  |  | 8 |  |
| AK066942 | 259_12_32 |  | TC253984 | 286_12_43 |  | TC133589 | 262_12_36 |  |  |  | 67 | Expressed protein |
| AK066480 | 146_24_104 |  | TC256019 | 215_24_326 |  | TC148944 | 275_24_194 |  |  |  | 14 | Hypothetical protein |
| AK066424 | 406_6_302 |  |  |  |  | TC148993 | 13_6_560 |  | TC281469 | 539_6_92 | 100 | RING zinc finger protein-like |
| AK066073 | 154_75_125 |  | TC236575 | 204_75_379 |  |  |  |  | TC293675 | 596_75_169 | 1 | Acetyl-coenzyme A synthetase |
| AK065998 | 108_72_28 |  | TC253336 | 232_69_101 |  | TC150526 | 760_72_308 |  |  |  | 4 | Hypothetical protein |
| AK065863 | 644_45_390 |  | TC255161 | 74_45_17 |  |  |  |  | TC287626 | 306_45_23 | 6 | Multidrug-resistance associated protein 1 |
| AK065729 | 398_9_62 |  | TC243618 | 239_9_191 |  | TC134511 | 147_9_291 |  |  |  | 50 | Hypothetical protein |
|  | 244_9_216 |  |  | 239_9_191 |  |  | 147_9_291 |  |  |  | 50 |  |
|  | 193_60_216 |  |  | 62_57_320 |  |  | 229_63_155 |  |  |  | 1 |  |
| AK065683 | 82_18_41 |  | TC243502 | 103_18_600 |  | TC153017 | 307_18_9 |  |  |  | 20 | Cell division protein kinase 8 |
| AK065578 | 470_120_117 |  | TC249752 | 503_117_110 |  |  |  |  | TC305318 | 318_117_159 | 1 | Transformer-2-like protein |
|  | 362_108_237 |  |  | 339_108_283 |  |  |  |  |  | 362_108_124 | 77 |  |
|  | 325_51_331 |  |  | 302_51_377 |  | TC139461 | 274_51_93 |  |  |  | 69 |  |
| AK065538 | 162_24_57 |  |  |  |  | TC139620 | 197_24_39 |  | TC287533 | 93_24_70 | 14 | Clathrin coat assembly protein AP47 |
| AK065240 | 112_21_17 |  |  |  |  | TC132139 | 231_21_13 |  | TC298549 | 354_21_28 | 83 | Arabinoxylan arabinofuranohydrolase isoenzyme |
| AK065176 | 333_12_179 |  | TC235016 | 413_12_202 |  | TC139184 | 466_12_203 |  |  |  | 67 | Phosphatidylinositol |
|  | 315_30_179 |  |  | 395_30_202 |  |  | 448_30_203 |  |  |  | 44 |  |
| AK065137 | 8_21_281 |  | TC251833 | 13_21_266 |  | TC147261 | 7_21_263 |  |  |  | 83 | Kelch-like ECH-associated protein 1 |
| AK065016 | 15_63_292 |  |  |  |  | TC134594 | 263_63_414 |  | TC287474 | 127_66_507 | 4 | Hydroxyproline-rich |
| AK064864 | 75_15_41 |  |  |  |  | TC138646 | 106_15_35 |  | TC288136 | 159_15_32 | 75 | Unknown protein |
| AK064792 | 281_99_98 |  | TC267323 | 259_99_98 |  |  |  |  | TC306152 | 268_99_98 | 72 |  |
|  | 276_15_187 |  |  | 254_15_187 |  |  |  |  |  | 263_15_187 | 100 |  |
| AK063846 | 171_12_19 |  | TC239301 | 99_12_12 |  |  |  |  | TC287762 | 164_12_12 | 67 | protein F12M16.29 |
| AK061109 | 35_12_98 |  | TC263378 | 53_12_68 |  |  |  |  | TC306071 | 79_12_66 | 33 | Hypothetical protein |
| AK061004 | 108_9_30 |  | TC269443 | 96_9_25 |  | TC151138 | 130_9_25 |  |  |  | 100 | Peptidylprolyl isomerase |
| AK060780 | 546_6_320 |  |  |  |  | TC134531 | 513_6_122 |  | TC311790 | 53_6_34 | 100 | Pelota (PEL1) |
|  | 440_6_426 |  |  |  |  |  | 513_6_122 |  |  | 53_6_34 | 100 |  |
| AK059720 | 301_33_27 |  | TC239546 | 86_33_74 |  | TC149822 | 139_33_102 |  |  |  | 10 | Hypothetical protein |
| AK059001 | 179_117_246 |  | TC269581 | 134_120_152 |  | TC142662 | 193_117_14 |  |  |  | 10 | Calyx protein |
|  | 170_126_246 |  |  | 134_120_152 |  |  | 184_126_14 |  |  |  | 7 |  |
| AK058988 | 139_69_294 |  | TC235910 | 265_69_267 |  |  |  |  | TC314670 | 272_69_84 | 45 | Calcium-binding protein-like |
| AK058880 | 106_51_4 |  | TC269547 | 203_51_4 |  | TC152057 | 83_51_420 |  |  |  | 50 | Lipase class 3-like |
| AK058513 | 94_24_26 |  |  |  |  | TC147191 | 616_24_26 |  | TC305089 | 645_24_26 | 57 | Neutral leucine aminopeptidase |
|  | 34_84_26 |  |  |  |  |  | 556_84_26 |  |  | 585_84_26 | 63 |  |
|  | 128_6_10 |  |  |  |  |  | 640_6_20 |  |  | 669_6_20 | 100 |  |
|  | 118_24_2 |  |  |  |  |  | 616_24_26 |  |  | 645_24_26 | 14 |  |
| AK058462 | 11_15_32 |  |  |  |  | TC150825 | 122_15_42 |  | TC282858 | 232_15_86 | 25 | Transporter associated with antigen |
| AK121850 | 86_18_51 |  | TC238796 | 102_18_57 |  | TC140406 | 84_18_58 |  |  |  | 40 | Kinase CK2 regulatory subunit |
| ^a^ Pre orf distance_uORF length_intercistronic distance  ^b^ Functional annotation based on “The UniProt Knowledgebase (UniProt)” database  Identifiers may not be unique among the tables as different combinations of uORFs were conserved.  Ribosomal rRNA genes have been removed. | | | | | | | | | | | | |
