# Supplementary material for: Conserved upstream open reading frames in higher plants
Source: BMC Genomics. 2008 Jul 31;9:361. doi: 10.1186/1471-2164-9-361 (PMC2527020; doi:10.1186/1471-2164-9-361)
Supplement: Additional file 5 — TRAN_TableS5. 'The uORFs predicted by uORFSCAN in 3 out of 3'. [file 1471-2164-9-361-S5.doc]

| Table S5. The uORFs predicted by uORFSCAN in 3 out of 3 | | | | | | | | | |
| --- | --- | --- | --- | --- | --- | --- | --- | --- | --- |
| Rice | |  | Wheat | |  | Barley | | Avg. A.A. similarity (%) | Putative function^b^ |
| Identifer | 5′-UTR^a^ |  | Identifer | 5′-UTR^a^ |  | Identifer | 5′-UTR^a^ |
| AK122166 | 338_18_606 |  | TC250897 | 2_18_164 |  | TC149838 | 233_18_254 | 20 | Translation initiation factor 3 |
| AK122131 | 322_9_28 |  | TC251213 | 242_9_30 |  | TC147542 | 253_9_31 | 100 | Chitin-inducible gibberellin-responsive |
| AK121850 | 86_18_51 |  | TC238796 | 102_18_57 |  | TC140406 | 84_18_58 | 40 | Kinase CK2 regulatory subunit |
| AK121122 | 743_21_34 |  | TC266483 | 118_21_70 |  | TC148472 | 179_21_69 | 14 | NF protein |
| AK120494 | 199_21_34 |  | TC256417 | 136_21_625 |  | TC134801 | 394_21_404 | 17 | Hypothetical protein F17M5.140 |
| AK119650 | 98_21_61 |  | TC247011 | 129_21_73 |  | TC148824 | 219_21_8 | 17 | MAP kinase MAPK2 |
| AK111887 | 244_21_108 |  | TC235829 | 289_21_48 |  | TC131138 | 232_21_48 | 67 | Calcineurin B protein |
| AK106310 | 547_72_149 |  | TC273695 | 366_72_174 |  | TC136869 | 1_72_168 | 21 | Hypothetical protein |
|  | 128_21_619 |  |  | 471_21_120 |  |  | 106_21_114 | 17 |  |
| AK104437 | 187_42_203 |  | TC266855 | 203_42_174 |  | TC133317 | 181_42_178 | 92 | RNA-binding protein cabeza |
| AK103631 | 328_24_87 |  | TC253392 | 188_24_308 |  | TC139875 | 224_24_589 | 13 | Hypothetical protein |
|  | 313_39_87 |  |  | 173_39_308 |  |  | 209_39_589 | 17 |  |
| AK103391 | 205_75_74 |  | TC269775 | 251_75_62 |  | TC134190 | 204_75_62 | 92 | Trehalose-6-phosphate phosphatase |
|  | 157_123_74 |  |  | 203_123_62 |  |  | 156_123_62 | 80 |  |
|  | 130_27_197 |  |  | 176_27_185 |  |  | 129_27_185 | 38 |  |
|  | 118_39_197 |  |  | 164_39_185 |  |  | 117_39_185 | 50 |  |
| AK103390 | 277_51_25 |  | TC236507 | 242_51_24 |  | TC132330 | 379_51_24 | 69 | Non-imprinted in Prader-Willi/Angelman syndrome region protein 2 |
| AK103140 | 271_36_1 |  | TC266113 | 212_36_407 |  | TC140479 | 182_36_1 | 73 | Hypothetical protein |
| AK103040 | 45_87_201 |  | TC254504 | 50_84_166 |  | TC148672 | 25_84_166 | 64 | Single myb histone 1 |
| AK102966 | 206_9_32 |  | TC247483 | 188_9_14 |  | TC142783 | 160_9_138 | 50 | Type 5 serine/threonine phosphatase 55 |
| AK102376 | 115_24_31 |  | TC237876 | 95_24_48 |  | TC133824 | 87_24_47 | 14 | Zinc finger (C3HC4-type RING finger) |
| AK102370 | 127_60_11 |  | TC255624 | 139_63_11 |  | TC133336 | 401_57_20 | 30 | Tubby-like protein 3 |
| AK102068 | 463_12_11 |  | TC243607 | 181_12_14 |  | TC136167 | 397_12_315 | 33 | Hypothetical protein |
| AK101684 | 158_21_12 |  | TC253407 | 137_21_33 |  | TC141318 | 104_21_33 | 50 | CCAAT-box transcription factor |
| AK101539 | 37_12_125 |  | TC251540 | 98_12_110 |  | TC140495 | 36_12_129 | 100 | CG11670-PA |
| AK101319 | 532_87_646 |  | TC271530 | 20_87_40 |  | TC142174 | 434_87_0 | 14 | Hypothetical protein F14F8_120 |
| AK101100 | 142_12_21 |  | TC263224 | 132_12_14 |  | TC132639 | 175_12_510 | 100 | Protein phosphatase 2A 55 kDa B |
| AK100440 | 246_81_195 |  | TC235293 | 210_78_152 |  | TC133630 | 180_78_153 | 31 | BZIP transcription factor, complete |
| AK100037 | 449_33_85 |  | TC234512 | 400_33_495 |  | TC134276 | 222_33_507 | 90 | SAC domain-containing protein |
| AK099852 | 906_9_2 |  | TC233509 | 103_9_218 |  | TC144509 | 159_9_177 | 50 | Hypothetical protein |
| AK099839 | 147_48_82 |  | TC237323 | 145_48_50 |  | TC140250 | 758_48_585 | 20 | MAP3K epsilon protein kinase |
| AK099745 | 136_21_245 |  | TC269480 | 129_21_140 |  | TC136177 | 22_21_245 | 17 | Glutamate receptor 3.2 |
| AK073985 | 101_12_101 |  | TC252583 | 179_12_900 |  | TC148772 | 149_12_82 | 67 | RNA-binding protein FUS |
| AK073303 | 67_9_142 |  | TC237149 | 75_9_113 |  | TC132556 | 81_9_139 | 100 | Hypothetical protein |
|  | 135_9_74 |  |  | 75_9_113 |  |  | 81_9_139 | 50 |  |
| AK072868 | 392_36_96 |  | TC247418 | 404_36_111 |  | TC139536 | 444_36_117 | 91 | Serine/threonine kinase |
|  | 377_51_96 |  |  | 389_51_111 |  |  | 429_51_117 | 81 |  |
|  | 338_90_96 |  |  | 347_93_111 |  |  | 387_93_117 | 53 |  |
|  | 269_39_216 |  |  | 278_39_234 |  |  | 318_39_240 | 83 |  |
|  | 259_195_70 |  |  | 268_198_85 |  |  | 308_198_91 | 65 |  |
|  | 249_27_248 |  |  | 258_27_266 |  |  | 298_27_272 | 75 |  |
| AK072649 | 100_192_117 |  | TC236348 | 79_192_117 |  | TC133316 | 76_192_93 | 87 | Ribosomal protein S6 kinase homolog |
| AK072244 | 124_15_222 |  | TC252797 | 107_15_14 |  | TC136383 | 66_15_390 | 25 | Hypothetical protein |
| AK072085 | 725_6_132 |  | TC253625 | 6_6_624 |  | TC150175 | 602_6_23 | 100 | RNA polymerase II termination |
|  | 683_6_174 |  |  | 6_6_624 |  |  | 602_6_23 | 100 |  |
|  | 469_6_388 |  |  | 6_6_624 |  |  | 602_6_23 | 100 |  |
|  | 397_78_388 |  |  | 491_78_67 |  |  | 229_81_321 | 1 |  |
| AK070766 | 144_15_65 |  | TC263230 | 129_15_44 |  | TC134132 | 121_15_44 | 75 | PG4 |
| AK070751 | 664_33_209 |  | TC240522 | 226_33_83 |  | TC142763 | 298_33_5 | 9 | F7N22.3 protein |
| AK069730 | 770_156_22 |  | TC246998 | 270_150_246 |  | TC132118 | 275_159_249 | 15 | Hypothetical protein |
|  | 412_153_383 |  |  | 270_150_246 |  |  | 275_159_249 | 47 |  |
| AK069726 | 120_78_82 |  | TC235568 | 119_78_74 |  | TC139583 | 107_78_72 | 80 | Hordeum vulgare mRNA for expressed sequence tag |
| AK069534 | 870_57_327 |  | TC236981 | 222_57_931 |  | TC139404 | 90_57_190 | 6 | Auxilin-like protein |
|  | 603_57_594 |  |  | 222_57_931 |  |  | 90_57_190 | 6 |  |
|  | 411_93_750 |  |  | 982_93_135 |  |  | 51_96_190 | 6 |  |
|  | 1068_96_90 |  |  | 982_93_135 |  |  | 51_96_190 | 3 |  |
| AK069526 | 737_87_60 |  | TC265553 | 757_87_62 |  | TC147034 | 740_87_62 | 54 | GAMYB-binding protein |
|  | 690_9_185 |  |  | 709_9_188 |  |  | 692_9_188 | 100 |  |
|  | 440_102_342 |  |  | 453_102_351 |  |  | 436_102_351 | 18 |  |
|  | 214_126_544 |  |  | 239_123_544 |  |  | 222_123_544 | 80 |  |
|  | 149_246_489 |  |  | 174_243_489 |  |  | 157_243_489 | 63 |  |
| AK069065 | 133_12_97 |  | TC266624 | 198_12_77 |  | TC132959 | 163_12_73 | 33 | RAD23-like protein |
| AK067412 | 222_84_49 |  | TC252944 | 247_81_102 |  | TC142664 | 123_84_118 | 19 | Protein kinase |
| AK067258 | 246_27_25 |  | TC247646 | 508_27_43 |  | TC140304 | 193_27_46 | 38 | Ankyrin-like protein |
| AK067156 | 1433_6_261 |  | TC238252 | 27_6_439 |  | TC140969 | 143_6_148 | 100 | Hypothetical protein |
| AK066952 | 437_57_119 |  | TC271435 | 169_57_23 |  | TC137456 | 357_57_231 | 9 | Arabidopsis thaliana genomic DNA |
|  | 392_39_182 |  |  | 153_39_57 |  |  | 222_39_384 | 8 |  |
| AK066942 | 259_12_32 |  | TC253984 | 286_12_43 |  | TC133589 | 262_12_36 | 67 | Expressed protein |
| AK066480 | 146_24_104 |  | TC256019 | 215_24_326 |  | TC148944 | 275_24_194 | 14 | Hypothetical protein |
| AK066307 | 1325_12_14 |  | TC264007 | 215_12_16 |  | TC149400 | 160_12_15 | 100 | RNA polymerase alpha subunit |
|  | 1232_6_113 |  |  | 109_6_128 |  |  | 60_6_121 | 100 |  |
| AK066145 | 178_12_58 |  | TC266262 | 149_12_73 |  | TC134484 | 154_12_231 | 67 | Protein F2E2.12 |
| AK065998 | 108_72_28 |  | TC253336 | 232_69_101 |  | TC150526 | 760_72_308 | 4 | Hypothetical protein |
| AK065729 | 398_9_62 |  | TC243618 | 239_9_191 |  | TC134511 | 147_9_291 | 50 | Hypothetical protein |
|  | 244_9_216 |  |  | 239_9_191 |  |  | 147_9_291 | 50 |  |
|  | 193_60_216 |  |  | 62_57_320 |  |  | 229_63_155 | 1 |  |
| AK065683 | 82_18_41 |  | TC243502 | 103_18_600 |  | TC153017 | 307_18_9 | 20 | Cell division protein kinase 8 |
| AK065585 | 126_15_34 |  | TC254095 | 64_15_42 |  | TC139863 | 34_15_48 | 100 | Monodehydroascorbate reductase |
| AK065578 | 325_51_331 |  | TC249752 | 302_51_377 |  | TC139461 | 274_51_93 | 69 | Transformer-2-like protein |
| AK065329 | 444_15_35 |  | TC238280 | 174_15_44 |  | TC147756 | 276_15_44 | 50 | Hypothetical protein F14M19.150 |
| AK065176 | 333_12_179 |  | TC235016 | 413_12_202 |  | TC139184 | 466_12_203 | 67 | Phosphatidylinositol 3 |
|  | 315_30_179 |  |  | 395_30_202 |  |  | 448_30_203 | 44 |  |
| AK065137 | 8_21_281 |  | TC251833 | 13_21_266 |  | TC147261 | 7_21_263 | 83 | Kelch-like ECH-associated protein 1 |
| AK063875 | 128_78_44 |  | TC238591 | 348_78_2 |  | TC133365 | 519_78_2 | 23 | Prokineticin 2 precursor |
| AK061004 | 108_9_30 |  | TC269443 | 96_9_25 |  | TC151138 | 130_9_25 | 100 | peptidylprolyl isomerase |
| AK060783 | 216_21_173 |  | TC247469 | 243_21_176 |  | TC132683 | 307_21_175 | 100 | Hypothetical protein |
| AK060523 | 173_123_185 |  | TC235416 | 201_126_157 |  | TC148319 | 211_120_163 | 68 | Hypothetical protein |
| AK060232 | 38_15_10 |  | TC273755 | 224_15_44 |  | TC135479 | 410_15_99 | 25 | SAM-dependent methyltransferase-like |
| AK059720 | 301_33_27 |  | TC239546 | 86_33_74 |  | TC149822 | 139_33_102 | 10 | Hypothetical protein |
| AK059394 | 1_9_26 |  | TC270230 | 46_9_128 |  | TC134188 | 182_9_266 | 50 | Small nuclear ribonucleoprotein |
| AK059001 | 179_117_246 |  | TC269581 | 134_120_152 |  | TC142662 | 193_117_14 | 10 | Calyx protein |
|  | 170_126_246 |  |  | 134_120_152 |  |  | 184_126_14 | 7 |  |
| AK058880 | 106_51_4 |  | TC269547 | 203_51_4 |  | TC152057 | 83_51_420 | 50 | Lipase class 3-like |
| ^a^ Pre orf distance_uORF length_intercistronic distance  ^b^ Functional annotation based on “The UniProt Knowledgebase (UniProt)” database  Identifiers may not be unique among the tables as different combinations of uORFs were conserved.  Ribosomal rRNA genes have been removed. | | | | | | | | | |
