# Supplementary material for: Conserved upstream open reading frames in higher plants
Source: BMC Genomics. 2008 Jul 31;9:361. doi: 10.1186/1471-2164-9-361 (PMC2527020; doi:10.1186/1471-2164-9-361)
Supplement: Additional file 8 — TRAN_TableS6. 'ClustalW alignment of uORFs identified by uORFSCAN in 5 out of 5 cereals and in Arabidopsis'. [file 1471-2164-9-361-S8.doc]

| **Table S6.** ClustalW alignment of uORFs identified by uORFSCAN in 5/5 cereals and in Arabidopsis | |
| --- | --- |
| Rice identifier | Alignment^a^ |
| AK106095 | AK106095_r_ORF_131_9_17 ML  TC265929_w_ORF_113_9_16 ML  TC148181_b_ORF_67_9_16 ML  TC288369_m_ORF_131_9_17 ML  TC102998_s_ORF_149_9_17 ML  ** |
| AK103391 | AK103391_r_ORF_205_75_74 MNCLHTCSDKKTLKKWFFIDKTVG  TC269775_w_ORF_251_75_62 MNFLHTCSDKKTLKKWFFIDKTVG  TC134190_b_ORF_204_75_62 MNFHHTCSDKKTLKKWFFIDKTVG  TC294011_m_ORF_215_75_75 MNCLHTCGDKKTLKKWFFIDKTVG  TC103599_s_ORF_106_75_378 MNCLHTCSDKKTLKKWFFIDKTVG  ** ***.**************** |
| AK100589_uORF1 | AK100589_r_ORF_240_9_334 MY  TC264559_w_ORF_201_9_317 MC  TC130707_b_ORF_228_9_318 MF  TC292591_m_ORF_286_9_320 MY  TC91317_s_ORF_260_9_329 MY  * |
| AK100589_uORF2 | AK100589_r_ORF_248_156_179 MESKGGKKKSSSSRSLMYEAPLGYSIEDVRPAGGVKKFQSAAYSNCAKKPS  TC264559_w_ORF_209_150_168 MESKGGKK-SSSSSSLMYEAPLGYSIEDVRPAGGAKKF-SAAYSNCAKKPS  TC130707_b_ORF_236_150_169 MESKGGKK-SSSSSSLMYEAPLGYSIEDVRPAGGAKKF-SAAYSNCAKKPS  TC292591_m_ORF_294_153_168 MESKGGKK-SSSSRSMMYEAPLGYSIEDVRPAGGVKKFQSAAYSNCAKKPS  TC91317_s_ORF_268_153_177 MESKGGKK-SSSSRSMMYEAPLGYSIEDVRPAGGVKKFQSAAYSNCAKKPS  ******** **** *:******************.*** ************ |
| AK100589_uORF3 | AK100589_r_ORF_296_108_179 -MYEAPLGYSIEDVRPAGGVKKFQSAAYSNCAKKPS  TC264559_w_ORF_254_105_168 -MYEAPLGYSIEDVRPAGGAKKF-SAAYSNCAKKPS  TC130707_b_ORF_281_105_169 -MYEAPLGYSIEDVRPAGGAKKF-SAAYSNCAKKPS  TC292591_m_ORF_336_111_168 MMYEAPLGYSIEDVRPAGGVKKFQSAAYSNCAKKPS  TC91317_s_ORF_310_111_177 MMYEAPLGYSIEDVRPAGGVKKFQSAAYSNCAKKPS  ******************.*** ************ |
| AK073303_uORF1 | AK073303_r_ORF_67_9_142 MP  TC237149_w_ORF_75_9_113 MP  TC132556_b_ORF_81_9_139 MP  TC305609_m_ORF_127_9_69 MI  TC102988_s_ORF_222_9_69 MI  * |
| AK073303_uORF2 | AK073303_r_ORF_135_9_74 MI  TC237149_w_ORF_75_9_113 MP  TC132556_b_ORF_81_9_139 MP  TC305609_m_ORF_127_9_69 MI  TC102988_s_ORF_222_9_69 MI  * |
| AK072868_uORF1 | AK072868_r_ORF_249_27_248 MQKDVLAC-  TC247418_w_ORF_258_27_266 MQKDVFAC-  TC139536_b_ORF_298_27_272 MQRDVFAC-  TC306591_m_ORF_444_27_564 MVK-IAGHL  TC102544_s_ORF_331_27_265 MQKDVLAC-  * : : . |
| AK072868_uORF2 | AK072868_r_ORF_259_195_70 MCLHARELPCEGIGRVASHISPSTTLHDIGTQEYI-QRLLHVLSHYGVRRGNSTIFLDHHLGGDG  TC247418_w_ORF_268_198_85 MCLHARELPCEGIGRVAAPVSALIDLDDTASQQHTTHLFFHVLLHNGVRRGISTIILDYHLGGDG  TC139536_b_ORF_308_198_91 MCLHARELPCEGIGRVAAPLSALIDLDDTASQHHTAHLFFHVLLHNGVRRGISTIILDYHLGGDG  TC306591_m_ORF_260_192_583 MWLHD-GVPCLEIGRIHKHSCTLLDLDDIGLQIYA-QQLPHAHTHTGAASCSSTIVSGFFLGGDG  TC102544_s_ORF_341_195_87 MCLHVEELPCEGLGRVAHHIDSLPALDDLAAQEYT-HLLLLVLPHNGVRCGGSTVFLDHHLGGDG  * ** :** :**: . *.* . * : : : . * *. **:. ...***** |
| AK072868_uORF3 | AK072868_r_ORF_269_39_216 MLESYLVR-ELAG  TC247418_w_ORF_278_39_234 MLESYLAR-ESAG  TC139536_b_ORF_318_39_240 MLESYLAR-ESAG  TC306591_m_ORF_768_39_228 -MRLWLPKPRYIL  TC102544_s_ORF_351_39_233 MLKSYLVR-DLAG  :. :* : |
| AK072868_uORF4 | AK072868_r_ORF_338_90_96 MT-LEHKSIYSACSMCSRTMGFDVATQPSS-  TC247418_w_ORF_347_93_111 MTPLHSSTQHTSSSMCFCTMGFDVASQPSS-  TC139536_b_ORF_387_93_117 MTPPRSTTQRTSSSMCFCTMGFDVASQPSS-  TC306591_m_ORF_576_93_366 MRWESYLEKGVLPKFTMLAM-LRLQKALLSR  TC102544_s_ORF_420_90_113 MT-LQLKSTRIFSYLCFRTMGFDVAAQPSS-  * : :* : : * |
| AK072868_uORF5 | AK072868_r_ORF_392_36_96 -MGFDVATQPSS  TC247418_w_ORF_404_36_111 -MGFDVASQPSS  TC139536_b_ORF_444_36_117 -MGFDVASQPSS  TC306591_m_ORF_633_36_366 MLRLQKALLSR-  TC102544_s_ORF_474_36_113 -MGFDVAAQPSS  : :: * . |
| AK072649 | AK072649_r_ORF_100_192_117 MNSGSTLASRVSCSSGNNGIFPPCLLPGFLGFPVVFVNFHLFKAIVRSPASSVVLGICGGRLP  TC236348_w_ORF_79_192_117 MYSGRTVASRVSCSSGNNGSFPPCLCPGFLGFPVVSVNFHLFKAIVRSPASSVVLGICGRSLP  TC133316_b_ORF_76_192_93 MYSGRTVASRVSCSSGNNGSFPPCLRPGFLGFPVVSVNFHLFKAIVRSPASSVVLGICGRSLP  TC305793_m_ORF_180_192_116 MNSGRTVASSVSCPAGNSGSFPPCLLPGFLGFPVVFVNFHLFKAIVRSPASSVVLGICGGSLP  TC93140_s_ORF_168_192_116 MNSGRTVASNVSCSAGNSGSFPPCLLPGFLGFPVIFVNFHLFKAIVRSPASSVILGICGGSLP  * ** *:** ***.:**.* ***** ********: *****************:***** ** |
| Rice identifier | Alignment^a^ |
| AK064792 | AK064792_r_ORF_276_15_187 MLCC  TC267323_w_ORF_254_15_188 MLCC  TC132983_b_ORF_253_15_-9 MLCC  TC306152_m_ORF_263_15_170 MLCC  TC107743_s_230_15_150 MLCC  **** |
| AK060523 | AK060523_r_ORF_173_123_185 MVLT-----PSPSPPPMLPKKLRALGPGLNPFAPFGMGNYYSSSR  TC235416_w_ORF_201_126_157 MVRR-RPSSSSTSSSPMLHKNLRALGPGLNPFAPFGMGNY---SR  TC148319_b_ORF_211_120_163 MVRR-RPSSS--SSSPMLHKNLRALGPGLNPFAPFGMGNY---SR  TC305149_m_ORF_255_129_195 MVYAPCRSSTPPSSSPMLHKNLRALGPGLNPLAPFGMGNY---SR  TC103609_s_ORF_240_129_212 MVYAPCRSSKPPSSSPMLHKNLRALGPGLNPFAPFGMGNY---TR  ** *..*** *:**********:******** :* |
| AK101100 | AK101100_r_ORF_142_12_21 MVS  AT1G51690.1_a_ORF_555_12_1160 MNI  * |
| AK066952_uORF1 | AK066952_r_ORF_365_66_182 MMKQRLILQMQVIR-LLMNVGT  AT3G13225.1_a_ORF_364_63_431 -MSWS-ILQLQAFWGLSSGCSS  *. ***:*.: * . .: |
| AK066952_uORF2 | AK066952_r_ORF_368_63_182 MKQRLILQMQVIR-LLMNVGT  AT3G13225.1_a_ORF_364_63_431 MSWS-ILQLQAFWGLSSGCSS  *. ***:*.: * . .: |
| AK066952_uORF3 | AK066952_r_ORF_503_51_59 -MIRSALEILLKKMLLP  AT3G13225.1_a_ORF_553_51_254 MQYKVSHSYTFSRSYN-  : : . :.: |
| AK119592 | AK119592_r_ORF_304_90_148 -------MKISTRLLWSTSFFRHKIAATIASSSSFL  AT3G01470.1_a_ORF_162_87_120 MGFCICPLESPARLLWSTSFFRHKIMIF--------  :: .:************* |
| AK100589 | AK100589_r_ORF_248_156_179 MESKGGKKKSSSSRSLMYEAPLGYSIEDVRPAGGVKKFQSAAYSNCAKKPS  AT3G02470.3_a_ORF_222_156_154 MESKGGKKKSSSSSSLFYEAPLGYSIEDVRPNGGIKKFKSSVYSNCSKRPS  ************* **:************** **:***:*:.****:*:** |
| AK103391_uORF1 | AK103391_r_ORF_176_30_148 MTSSQVFLC  AT4G22590.1_a_ORF_254_30_137 MISFQVTYF  * * ** |
| AK103391_uORF2 | AK103391_r_ORF_205_75_74 MNCLHTCSDKKTLKKWFFIDKTVG  AT4G22590.1_a_ORF_283_75_63 MDSSTTSSDKKTLKRWFFIDKRVG  *:. *.*******:****** ** |
| AK069534 | AK069534_r_ORF_813_9_432 MI  AT4G12770.1_a_ORF_41_9_108 ML  *: |
| AK069526_uORF1 | AK069526_r_ORF_214_126_544 MEYTLYTTSSSVLHISLLEEVLGWRFSLYGDFLVISFVNCT  AT4G19110.2_a_ORF_255_126_527 MEQVFVWPSCYHYRLFSFQEALDWRFLVRSDFLVGSFVNCT  ** .: .*. :: ::*.*.*** : .**** ****** |
| AK069526_uORF2 | AK069526_r_ORF_690_9_185 MA  AT4G19110.2_a_ORF_603_9_296 ML  * |
| AK069526_uORF3 | AK069526_r_ORF_820_36_28 MSLVHNRALLE-  AT4G19110.2_a_ORF_398_36_474 M-IFRGRCEANF  * :.:.*. : |
| AK072868 | AK072868_r_ORF_338_90_96 MTLEHKSIYSACSMCSRTMGFDVATQPSS-  AT5G58380.1_a_ORF_11_87_295 MTFNF--VFISSSSSSSVFSSIFVGKPRKK  **::. :: :.* .* .:. .. :* . |
| AK060523_uORF1 | AK060523_r_ORF_173_123_185 -MVLTPSPSPPPMLPKKLRALGPGLNPFAPFGMGNYYSSSR  AT5G07840.1_a_ORF_289_117_250 MLVFSSLSMTPVVIPQNLRVFGPGLNPSFPYCIANHFP---  :*::. . .* ::*::**.:****** *: :.*::. |
| AK060523_uORF2 | AK060523_r_ORF_206_90_185 ----MLPKKLRALGPGLNPFAPFGMGNYYSSSR  AT5G07840.1_a_ORF_313_93_250 MTPVVIPQNLRVFGPGLNPSFPYCIANHFP---  ::*::**.:****** *: :.*::. |
| AK067412 | AK067412_r_ORF_222_84_49 -MRAVVKRRRGGERGRCCGYWRSGASCD  AT5G50180.1_a_ORF_357_84_79 MLAIYLSLLFSSLSCELSNLHRYKSRK-  : :. .. . .. * : |
| AK102277 | AK102277_r_ORF_228_117_150 MHQRLHGWNKSTSMLRDGFGVKYSGFLHIRPCGFCRGD  AT1G68550.1_a_ORF_309_96_95 MRLRPKRTCSSVEVFG-GFHIKQQKFSFF----IVR--  *: * : .*..:: ** :* . * .: : * |
| ^a^ Identifier_code_ORF_pre-orf distance_orf length_intercistronic distance  code  r = rice  w = wheat  b = barley  m = maize  s = sorghum  a = Arabidopsis | |
