# Supplementary material for: Conserved upstream open reading frames in higher plants
Source: BMC Genomics. 2008 Jul 31;9:361. doi: 10.1186/1471-2164-9-361 (PMC2527020; doi:10.1186/1471-2164-9-361)
Supplement: Additional file 9 — uORFSCAN. 'uORFSCAN program'. [file 1471-2164-9-361-S9.zip › uORFSCAN/MANUAL.doc]

uORFSCAN MANUAL v1.0

System requirements

The uORFSCAN program was developed using the JAVA SE (JDK version 1.5.0_02), and has been tested in a Windows XP (SP2) environment.

uORFSCAN uses ClustalW version 1.83 and BioJava version 1.4

Windows setup

Download and install Java:

<http://java.sun.com/javase/downloads/index.jsp>

Set CLASSPATH variable for JDK and JRE in “Environment Variables” through the “System” option of the “Control Panel”.

For example, it might look like this:

C:\ProgramFiles\Java\jdk1.5.0_05\bin;C:\Program Files\Java\jre1.6.0_05\bin

Don’t add delimiter at the end of list. Reboot computer to initialise the variables.

# Input data format

Input data should be placed in folder “inputs”. Each data file containing sequences must be in a similar format to FASTA with the exception that the sequence should be on a single line. Data files must have the “txt” extension.

Starting the program

Goto command line, locate uORFSCAN directory, and type:

java –jar uORFSCAN.jar

Output data

Identified uORFs results are placed in folder “uORFs”. Alignments of uORFs are placed in folder “alignments”. Summary of results placed in folder “predictions”. The data in summary.txt is tab delimited, and may be imported into a database (e.g., Microsoft Access).

Reporting errors

E-mail [mgktran@hotmail.com](mailto:mgktran@hotmail.com) and attach input files and error file.
